# Genetic Architecture of Intrinsic Antibiotic Susceptibility

**DOI:** 10.1371/journal.pone.0005629

**Published:** 2009-05-20

**Authors:** Hany S. Girgis, Alison K. Hottes, Saeed Tavazoie

**Affiliations:** Lewis-Sigler Institute for Integrative Genomics and Department of Molecular Biology, Princeton University, Princeton, New Jersey, United States of America; Baylor College of Medicine, United States of America

## Abstract

**Background:**

Antibiotic exposure rapidly selects for more resistant bacterial strains, and both a drug's chemical structure and a bacterium's cellular network affect the types of mutations acquired.

**Methodology/Principal Findings:**

To better characterize the genetic determinants of antibiotic susceptibility, we exposed a transposon-mutagenized library of *Escherichia coli* to each of 17 antibiotics that encompass a wide range of drug classes and mechanisms of action. Propagating the library for multiple generations with drug concentrations that moderately inhibited the growth of the isogenic parental strain caused the abundance of strains with even minor fitness advantages or disadvantages to change measurably and reproducibly. Using a microarray-based genetic footprinting strategy, we then determined the quantitative contribution of each gene to *E. coli*'s intrinsic antibiotic susceptibility. We found both loci whose removal increased general antibiotic tolerance as well as pathways whose down-regulation increased tolerance to specific drugs and drug classes. The beneficial mutations identified span multiple pathways, and we identified pairs of mutations that individually provide only minor decreases in antibiotic susceptibility but that combine to provide higher tolerance.

**Conclusions/Significance:**

Our results illustrate that a wide-range of mutations can modulate the activity of many cellular resistance processes and demonstrate that *E. coli* has a large mutational target size for increasing antibiotic tolerance. Furthermore, the work suggests that clinical levels of antibiotic resistance might develop through the sequential accumulation of chromosomal mutations of small individual effect.

## Introduction

Antibiotic tolerance, the decreased efficacy of antimicrobial agents in eliminating infections, is a serious and worsening global problem in human health [1], [2]. During the long history of chemical warfare between microbes, the genomes of many bacteria have evolved to encode multiple counter-measures [3]. Moreover, a level of antibiotic tolerance that allows some bacteria to survive an initial exposure gives the population the opportunity to accumulate mutations, leading to higher levels of tolerance and potentially to full clinical resistance [4]. Much of the literature on antibiotic resistance focuses on tolerance to the high antibiotic levels typically used in a clinical setting (see [5] for an exception).

Even in clinical practice, however, bacteria commonly experience sub-inhibitory drug concentrations, which may be capable of reducing the growth rate but are lower than the minimum inhibitory concentration (MIC). The cyclical dosing regimen for most antibiotics, for example, may cause the drug's plasma concentration to approach the MIC for short intervals during treatment. Furthermore, micro-niches within the host, such as epidermis, lungs, and joints, may attain significantly lower drug concentrations than the plasma [6]. Finally, patient non-compliance with the prescribed frequency and duration of antibiotic use can allow plasma levels to fall below the MIC. In such circumstances, selection for more tolerant variants is strong.

Outside clinical settings, environments containing antibiotics, especially at sub-inhibitory concentrations, abound. Soil contains numerous antibiotic-producing species [7], which generate compounds with roles in killing competitors as well as in inter- and intra-species signaling [8]. Antibiotics also enter the soil through the use of manure from livestock whose feed was supplemented with antibiotics [9], and wastewater can contain multiple drugs at concentrations in the range of ng/L, even after treatment [10]. The rise in environmental antibiotic levels resulting from the widespread use of antibiotics has selected for resistant strains in both soil [11] and aqueous [12] environments.

Diverse mechanisms including drug target modification, enzymatic drug inactivation, and intracellular drug concentration reduction can lead to antibiotic resistance [13], [14], [15]. A variety of sources, such as lateral gene transfer and chromosomal mutations, can provide the underlying genetic changes, and clinically resistant strains often contain multiple alterations. The plasmids, transposons, and mobile chromosome cassettes that contribute to antibiotic resistance, including methicillin resistance, in *Staphylococcus aureus* are examples of well-studied extrinsic elements that confer antibiotic resistance [16]. Similarly, *E. coli* strains can receive contributions to quinolone resistance from *gyrA* and *parC* mutations as well as a reduction in outer membrane porins and an increase in drug efflux pump activity [17]. Cases where multiple mutations work collectively to give resistance, while of clear importance, are not well understood, in part because the range of mutations that can increase a particular bacterium's antibiotic tolerance is rarely known.

The effect of mutation in altering genetic programs and modulating susceptibility, however, is starting to receive attention. Multiple groups have assayed the drug susceptibility of bacteria containing families of simple genetic perturbations such as single gene deletions or transposon insertions [18], [19], [20], [21], [22]. These studies found that large numbers of genes influence drug susceptibility, including many whose dominant function is not resistance. Moreover, while some genes, such as those that code for drug efflux pumps, contribute to resistance to a wide range of compounds, many others are drug or drug class specific.

Most previous work focused on identifying genes whose removal changes the MIC by some predetermined threshold, usually 2-fold. The increasing number of examples of multiple genetic perturbations combining to confer high levels of resistance [23], [24], however, suggests that mutations of even mild individual benefit are of potential clinical relevance. As such, we decided to take a complementary approach to identifying mutations that alter antibiotic susceptibility.

Rather than testing MIC directly, we searched a library of transposon-mutagenized cells [25] for those with a competitive advantage at drug concentrations that moderately reduced the parental strain's growth rate. Competitive growth of the mutants for multiple generations caused the abundance of strains with even minor fitness advantages or disadvantages to change measurably and reproducibly. Applying microarray-based genetic footprinting [25] to the resulting population allowed us to identify and quantify the genetic determinants contributing to both antibiotic sensitivity and tolerance.

The experimental conditions proved to be particularly conducive to finding mutants fitter than the wildtype in the presence of antibiotics, enabling us to find many genes whose inactivation increases antibiotic tolerance. Analysis of individual mutants revealed a non-perfect correlation between a strain's fitness advantage in the conditions of the selection and its MIC in similar conditions. Explanations for and implications of the variations are discussed.

## Results and Discussion

### Selection of Transposon Insertion Mutants under Sub-inhibitory Antibiotic Exposure

**Figure 1:**
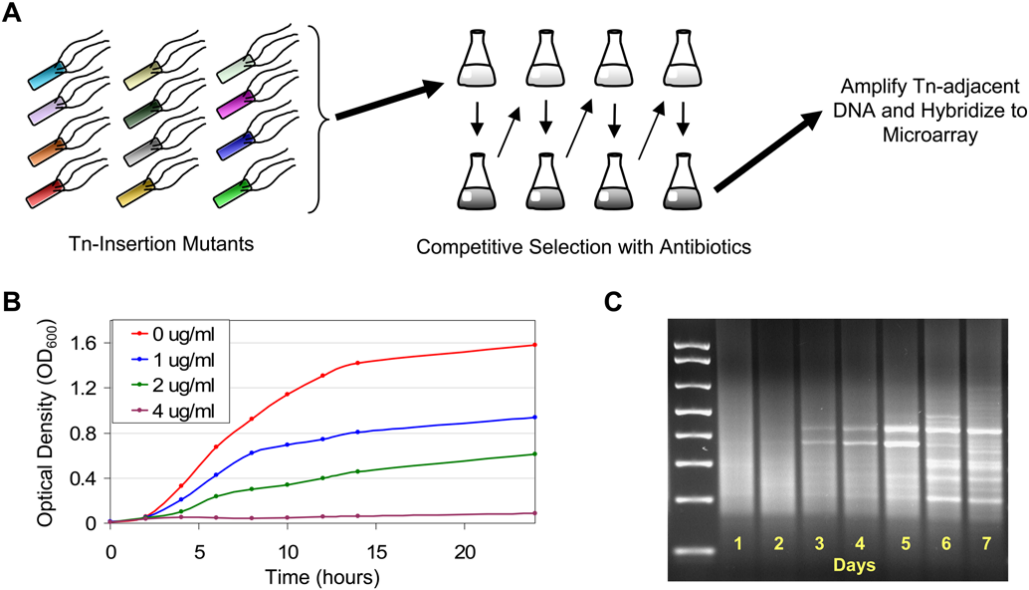
Overview of experimental protocol. (A) An aliquot of a library containing ∼5×10^5^ mutants each with a single transposon insertion [25] was taken from frozen stock, grown overnight in LB, pelleted, washed, and resuspended at 2% inoculum in fresh M9-media containing an antibiotic at the chosen concentration (Table 1). Each day, an aliquot was frozen, and 2% of the culture was transferred to fresh media to continue the selection. Genetic footprinting was performed on frozen samples to amplify the region of genomic DNA adjacent to the transposon in each of the mutants [25]. DNA was subsequently labeled and hybridized with a reference of labeled genomic DNA to spotted microarrays [25]. (B) Dose response curves were used to select drug concentrations. For each antibiotic, fresh media containing various drug concentrations was inoculated with overnight culture of the wild-type strain. Growth was monitored using OD_600_ readings. Shown are the curves for amikacin; curves for all other antibiotics are in Figure S1. Typically, we selected moderately inhibitory drug concentrations that reduced the growth after 14 hours by 30–50%. (C) Separation of DNA on an agarose gel provided a qualitative depiction of the population diversity after each day of selection. Shown are the amplified Tn-adjacent DNA from all seven days of one of the ampicillin selections. Selections performed without antibiotic showed no discernable banding pattern (Figure S2). Gel images for all selections with antibiotics are in Figure S3.

To characterize the contribution of all genetic loci to antibiotic tolerance, we exposed a collection of mutants, each with a single transposon insertion, to sub-inhibitory drug concentrations (Figure 1). We chose 17 antibiotics that possess a range of mechanisms of action (Table 1). These drugs inhibit various cellular functions including the synthesis of proteins, nucleic acids, folic acid, and cell wall [26], [27]. As intermediate antibiotic concentrations are typically the most selective for resistance [28], during the selections, we used antibiotic concentrations that impaired but did not completely inhibit the growth of the wild-type strain (Table 1). Using a sensitive microarray-based strategy [25], we then quantified the change in prevalence of different transposon insertion mutants in parallel.

**Table 1:** Information related to each antibiotic used in this study.

Name	Code	Dose (μg/ ml)	Day	# Samples	Class	Cellular Target	Bactericidal or Bacteriostatic
Ampicillin	AMP	3	2	3	β-lactam	Cell wall biosynthesis	Bactericidal
Amikacin	AMK	1	4	2	Aminoglycoside	Protein synthesis, 30S	Bactericidal
Bleomycin	BLM	1	2	2	Peptide	Nucleic acid	Bacteriostatic
Cefoxitin	FOX	1	3	2	β-lactam	Cell wall biosynthesis	Bactericidal
Doxycycline hyclate	DOX	0.5	2/3*	3	Tetracycline	Protein synthesis, 30S	Bacteriostatic
Erythromycin	ERY	2	3	2	Macrolide	Protein synthesis, 50S	Bacteriostatic
Fusidic acid	FUS	180	4	2		Protein synthesis, 50S	Bacteriostatic
Gentamycin	GEN	0.1	2	2	Aminoglycoside	Protein synthesis, 30S	Bactericidal
Lomefloxacin	LOM	0.05	2/3*	3	Quinolone	DNA gyrase	Bactericidal
Nalidixic acid	NAL	4	2	2	Quinolone	DNA gyrase	Bactericidal
Nitrofurantoin	NIT	4	3	2	Nitroheterocyclic	Multiple Targets	Bactericidal
Piperacillin	PIP	1	4	2	β-lactam	Cell wall biosynthesis	Bactericidal
Streptomycin	STR	3	2	3	Aminoglycoside	Protein synthesis, 30 S	Bactericidal
Sulfamonomethoxine	SLF	0.5	2/3*	2	Sulfonamide	Folic acid biosynthesis	Bacteriostatic
Tetracycline	TET	0.25	2	2	Tetracycline	Protein synthesis, 30S	Bacteriostatic
Tobramycin	TOB	0.25	2	2	Aminoglycoside	Protein synthesis, 30S	Bactericidal
Trimethoprim	TRM	0.5	4	2	DHFR Inhibitor	Folic acid biosynthesis	Bacteriostatic

* Two samples from day 2 and one from day 3 were hybridized and analyzed.

The final distribution was compared to both the original, unselected library and to the library selected in the same media without antibiotics (See Materials and Methods). Comparison to populations grown in M9 media without antibiotics allowed media and antibiotic effects to be distinguished. As some external stresses such as low-level antibiotic exposure can ameliorate the deleterious nature of some mutations [29], the abundance of slow growing mutants may increase in cultures with antibiotics compared to cultures without antibiotics. Comparison to the unselected library allowed the identification of insertion locations whose prevalence had increased or decreased during the experiment and reduced the chances of classifying slow-growing mutants as less susceptible. Dataset S1 lists the loci whose disruption caused a significant fitness change in each of the drugs tested. Heatmaps displaying the data for loci whose disruptions were significant in individual drugs and classes of drugs are in Figures S4–[Supplementary-material pone.0005629.s006] [Supplementary-material pone.0005629.s007] [Supplementary-material pone.0005629.s008] [Supplementary-material pone.0005629.s009] [Supplementary-material pone.0005629.s010] [Supplementary-material pone.0005629.s011] S11.

### Validation of Global Fitness Profiling

Comparison to the literature indicates that we found many genes whose removal is known to increase susceptibility (See Tables S1–[Supplementary-material pone.0005629.s014] [Supplementary-material pone.0005629.s015] S4). However, attempts to set significance thresholds that captured all such genes resulted in an unacceptable number of false positives. As shown later in this work, some mutations confer such a high degree of fitness in particular antibiotics that strains possessing these mutations rapidly take over the population. The existence of such highly fit mutants makes it difficult to distinguish neutral transposon insertion locations from some with a slightly deleterious effect because mutants with either are quickly lost from the population. Consequently, when selecting genes for additional study, an emphasis was placed on those whose removal increases antibiotic tolerance.

**Figure 2:**
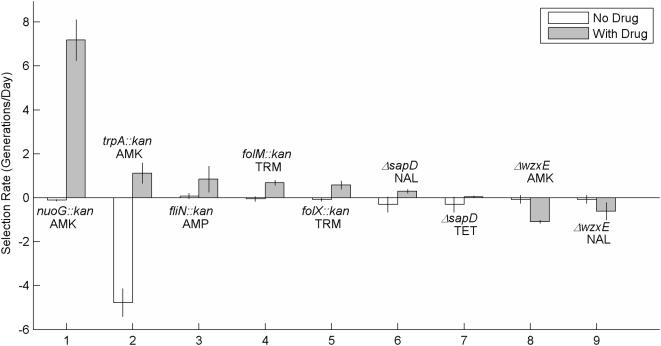
Selection rates during direct competitions. Selection rates (generations/day) were calculated as (log_2_(A(t_1_)/A(t_0_))−log_2_(B(t_1_)/B(t_0_)))/(t_1_−t_0_) [87]. A(t_0_) and B(t_0_) are, respectively, the mutant and the wild-type population sizes at t_0_, the beginning of the competition, and A(t_1_) and B(t_1_) are the mutant and the wild-type population sizes at t_1_, the end of the competition. Shown are the average and standard deviation of three repetitions. The selection rate for the *trpA::kan* mutant in amikacin was calculated after two days of enrichment to correspond with the samples hybridized. The *trpA::kan* strain's reliance on tryptophan from lysed wild-type cells prevents the mutant from taking over the culture, and during additional transfers, the wild-type strain showed a competitive advantage. Selection rates for other strains were insensitive to the competition duration.

To quantify the strength of the identified loci, we subjected the wild-type parental strain to direct competition with mutant strains in conditions that mimicked those of the selection (See Materials and Methods and Text S1). The mutants tested behaved as expected from the *en mass* library selections (Figure 2).

We also determined the MICs for a representative set of individual mutants lacking genes judged to have a significant role in antibiotic susceptibility. The relative efficiency of our MIC measurement method (see Text S1) allowed us to analyze a larger set of strains than would have been possible using only the more labor-intensive direct competitions. As the initial selection process did not require a change in MIC, the MICs provide complementary information about each mutation's effect, and we did not expect all beneficial (or deleterious) disruptions to cause an increase (or decrease) in MIC. The most common result was a change in MIC of 1.5 fold (the smallest change detectable with our technique) in the expected direction—an increase (or decrease) for a locus whose disruption was beneficial (or deleterious). We observed increases up to ∼5-fold and decreases down to ∼7-fold (Tables S5–[Supplementary-material pone.0005629.s018] S7).

### Genes Exert Class-Specific Effects on Antibiotic Susceptibility

**Figure 3:**
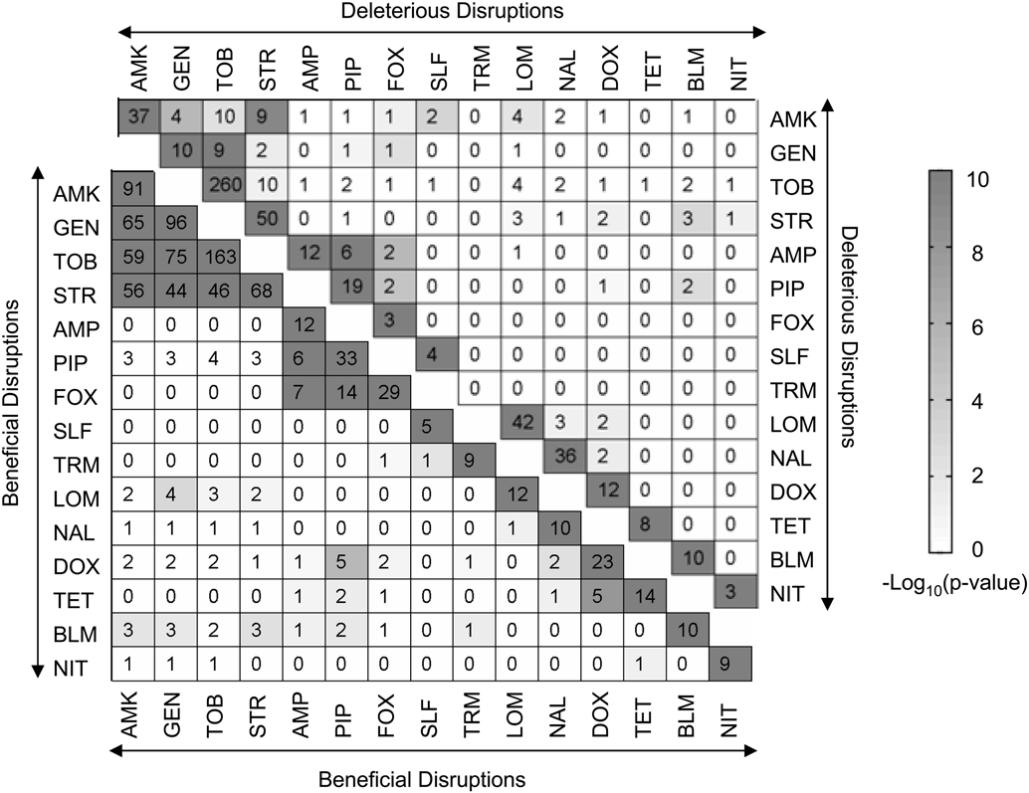
Overlap between genes influencing fitness in partially inhibitory concentrations of different antibiotics. Squares on the main diagonals indicate the number of genes whose disruption caused a significant fitness effect (See Materials and Methods). Genes causing general changes in antibiotic susceptibility (Figure 7) were excluded. The lower left (upper right) triangle reports on genes whose disruption was beneficial (deleterious) to *E. coli* in the presence of the indicated antibiotic. Off-diagonal squares indicate how many genes caused significant fitness changes in both antibiotics when disrupted. The shading shows the likelihood of an overlap of the indicated size or larger occurring by chance and was calculated using the hypergeometric distribution. P-values were corrected for multiple testing. Erythromycin and fusidic acid are not shown as the only genes whose disruption affected fitness caused general changes in susceptibility.

Excluding the 30 genes identified as having general roles in antibiotic susceptibility (see below), only antibiotics of the same class have a substantial number of beneficial or deleterious transposon insertion locations in common (Figure 3). Some functional classes of antibiotics, particularly the β-lactams and the aminoglycosides, have strong class-specific signatures, while other classes, such as the quinolones, have few loci in common. The number of loci impacting growth at the sub-inhibitory drug concentrations tested varied greatly from the low hundreds for the aminoglycosides down to zero for fusidic acid and erythromycin, inhibitors of the 50S subunit of the ribosome to which *E. coli* is intrinsically resistant [30]. The mutational target size observed likely depends on both the antibiotic concentration assayed and the structure of *E. coli*'s cellular network. For example, redundant pathways may mask the contribution of some genes to antibiotic tolerance.

**Figure 4:**
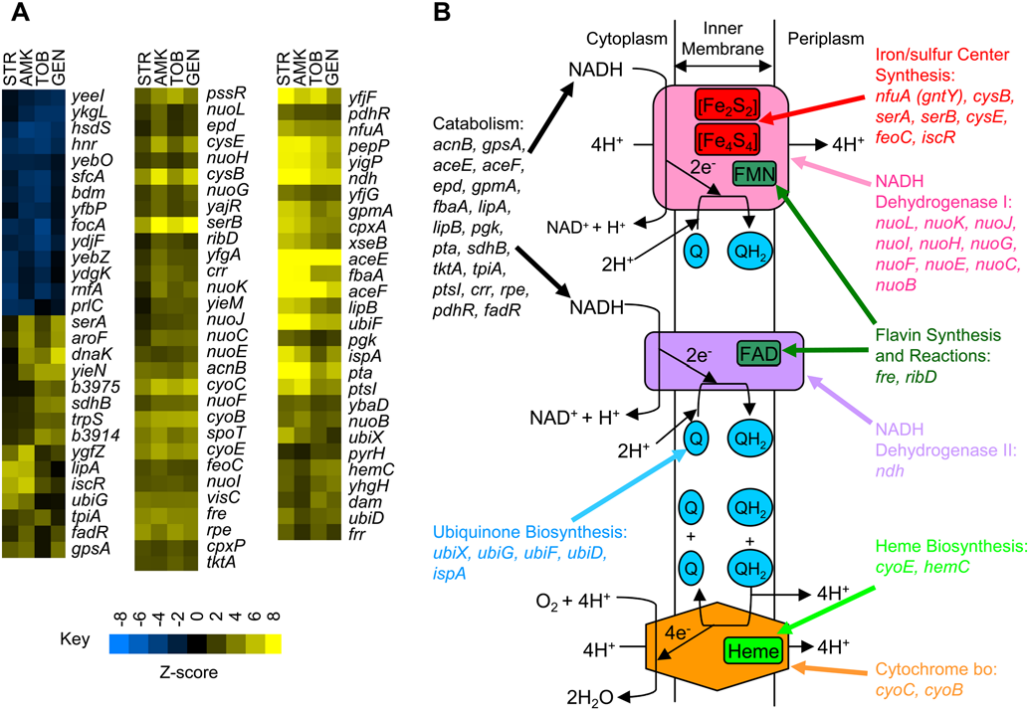
Disruption of electron transport and oxidative respiration reduces susceptibility to aminoglycosides. (A) The heatmaps, in which hierarchical clustering was used to order both the genes and the drugs [84], show loci whose disruption changed susceptibility to all four aminoglycosides tested (See Materials and Methods). Table S1 lists the genes with annotations. (B) Of the 73 transposon insertions regions identified as beneficial in all four aminoglycosides, the 48 shown are expected to reduce Fenton reaction-based oxidative damage. Following exposure to lethal concentrations of bactericidal antibiotics, the oxidative electron transport chain depletes the NADH pool, generating high levels of superoxide, which removes iron from iron-sulfur clusters [33]. The free iron subsequently generates hydroxyl radicals through the Fenton reaction [33]. Removal of key catabolic enzymes should shrink the NADH pool and reduce the flux through the electron transport chain. The media used lacks cysteine, the sulfur donor for iron-sulfur center synthesis [88], so disruption of cysteine biosynthesis should reduce the availability of sulfur for iron-sulfur centers. The iron-sulfur center synthesis genes shown are not specific for NADH dehydrogenase I, and their disruption should reduce the number of iron-sulfur clusters throughout the cell. Q: ubiquinone; FMN: flavin mononucleotide; FAD: flavin adenine dinucleotide

**Figure 5:**
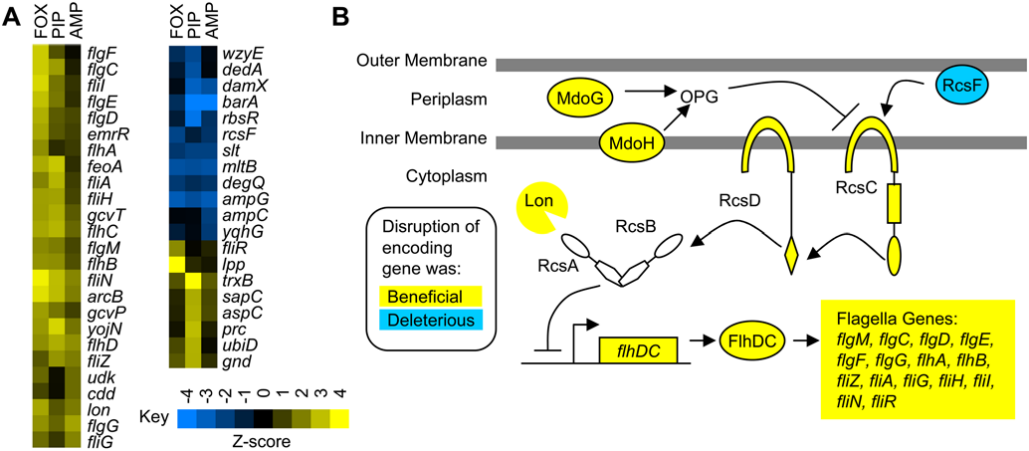
Reduced flagella synthesis is advantageous in β-lactams. (A) The heatmaps shows loci whose disruption changed susceptibility to all three β-lactams tested (See Materials and Methods). Hierarchical clustering was used to order both genes and drugs [84]. Table S2 lists the genes with annotations. (B) Both transposon insertions that disrupt genes that encode flagella components as well as insertions that indirectly reduce flagella synthesis by activating the Rcs system are beneficial. The core components of the Rcs system are RcsC, a hybrid sensor kinase, RcsD, a histidine phosphotransferase, and RcsB, a DNA-binding response regulator [89]. Other components are RcsF, a lipoprotein that activates RcsC [90], [91], and RcsA, a transcription factor that forms a heterodimer with RcsB [92]. Together, RcsA and RcsB repress transcription of *flhDC*, the master regulator of flagella synthesis [51]. RcsA is a target of the Lon protease [93], and insertions in *lon*, which stabilize RcsA, are beneficial. RcsC and RcsD both transfer phosphate to as well as remove phosphate from RcsB, resulting in higher activation of the Rcs system in *rcsC* or *rcsD* mutants than in wildtype [90], [94]. Insertions in *mdoG* and *mdoH*, which encode proteins that synthesize osmoregulated periplasmic glucans (OPGs), reduce motility by activating the Rcs system [25]. The beneficial effects of *mdoG*, *modH*, and *rcsC* disruptions are not limited to β-lactams (Figure 7).

**Figure 6:**
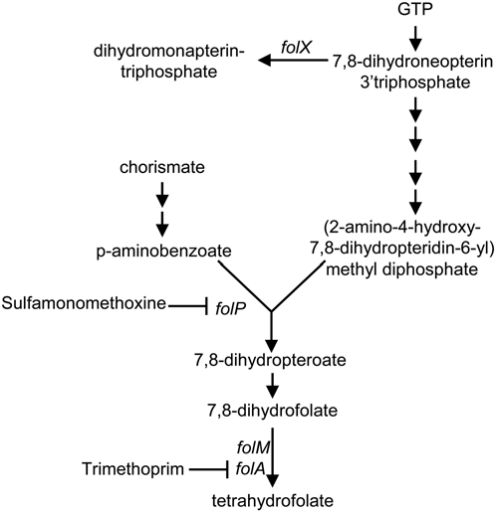
Genetic and chemical perturbations of the folate biosynthesis pathway. Sulfamonomethoxine inhibits FolP, a dihydropteroate synthase [95]; trimethoprim inhibits FolA, the cell's main dihydrofolate reductase (DHFR) [56]. FolM, which also acts as a DHFR, is not inhibited by trimethoprim [58]. Mutants lacking *folM* or *folX* are less sensitive to both to trimethoprim and sulfamonomethoxine.

The observation that disruptions caused increases in fitness during drug exposure is characteristic of antagonistic pleiotropy [31], which reflects an evolutionary tradeoff for increased drug tolerance at the consequence of other traits. A large number of disruptions fell into this category (336), suggesting that the bacterium contains a large mutational target size for increasing drug tolerance. To elucidate the pathways and mechanisms contributing to susceptibility and tolerance, we analyzed the identified loci for three drug classes – the aminoglycosides (Figure 4), the β-lactams (Figure 5), and the folic acid biosynthesis inhibitors (Figure 6) – in more detail. In each case, the data indicate the major pathways involved and the many ways that mutations can modulate those pathways.

### Mutations Beneficial in the Presence of Aminoglycosides

Excluding loci with a general effect on antibiotic tolerance, disruptions of 73 loci were beneficial in all four aminoglycosides tested while disruptions of 14 loci were deleterious (Figure 4A). Using an aerobic environment – a condition where many antibiotics, especially aminoglycosides, are particularly effective [32] – likely increased the number and types of beneficial mutations identified. The set of beneficial disruption loci is enriched for genes with products involved in quinone binding (p = 1.7×10^−15^), ubiquinone biosynthesis (p = 1.1×10^−5^), glycolysis (p = 1.5×10^−7^), L-serine biosynthesis (p = 1.2×10^−3^), and cytochrome-c oxidase activity (p = 1.2×10^−3^). Additionally, the set is enriched for genes down-regulated in response to oxygen depravation (p = 3.5×10^−16^). Forty-eight of the beneficial mutations likely reduce the number of harmful hydroxyl radicals created by the Fenton reaction, part of a death-inducing chain reaction triggered by bactericidal antibiotics that includes NADH depletion, superoxide generation, and iron-sulfur center destabilization [33] (Figure 4B). Kohanski et al. [34] found that a set of *E. coli* strains with similar mutations had increased growth in the presence of gentamycin. Similarly, Schurek et al. [22] found that mutants of *Pseudomonas aeruginosa* with disruptions in homologous genes had increased tobramycin resistance

To understand the magnitude of the tolerance changes, we competed a *nuoG::kan* strain with the wild-type strain. While the *nuoG::kan* strain was at a slight disadvantage in the media without drugs, in the presence of amikacin, the mutant rapidly took over the culture (Figure 2). In contrast, the *nuoG::kan* strain and other mutants that perturbed the pathway leading to death from Fenton reaction-derived hydroxyl radicals had at most modest (less than 3-fold) increases in MICs and frequently failed to exhibit any increase in MIC (Table S5). Since oxidative respiration processes are more active in the shaken cultures used for the competitions than in static MIC-measurement plates, the fitness effect of removing genes that contribute to the Fenton pathway likely depends strongly on the oxygen level.

Disruptions expected to interfere with the death pathway mediated by the Fenton reaction were widely beneficial only in selections with aminoglycosides, even though all bactericidal antibiotics are thought to trigger the pathway [33]. Furthermore, since the selections were performed at antibiotic concentrations that reduced, but did not completely inhibit, the wild-type strain's growth, observing genetic interactions with a putative death process was initially puzzling.

An explanation for the apparent paradox came from the observation that during the enrichments in amikacin and streptomycin, disruptions in tryptophan biosynthesis genes that should have been lethal in the growth media, which lacked tryptophan, were strongly beneficial. We confirmed that a *trpA::kan* mutant does not grow in the media, with or without antibiotics, and that when *trpA::kan* and wild-type strains compete without antibiotics, the wild-type strain rapidly takes over (Figure 2). When *trpA::kan* and wild-type strains compete in the presence of amikacin, however, the *trpA::kan* strain remains an appreciable part of the population (Figure 2). Presumably, a portion of the wild-type cells are dying, lysing, and releasing enough tryptophan to support the *trpA::kan* mutant. Thus, in the aminoglycoside enrichments, although the overall population was growing, individual cells were dying. The existence of similar concentration regimes for other bactericidal drugs remains an interesting area for future inquiry.

Several beneficial disruptions that likely reduce NADH accumulation and lower the metabolic flux through the Fenton reaction may also provide secondary benefits. First, disruption of electron transport reduces the uptake of aminoglycosides [35]. Second, mutations that keep cAMP-CRP (cyclic AMP bound to the CRP transcription factor) levels low reduce the transcription of the cAMP-CRP regulon, which has been suggested to include transporters with affinity for aminoglycosides [36]. Disruptions of both *cyaA*, which encodes adenylate cyclase, and *crp* were beneficial during the aminoglycoside selections, and a *cyaA::kan* mutant has a higher MIC than the wildtype in three of the four aminoglycosides tested (Table S5). (*crp* does not appear in Figure 4 due to lack of data in gentamycin and tobramycin; *cyaA* is not in Figure 4 because its deletion was generally advantageous.) Transposon insertions in *ptsH*, *ptsI*, and *crr*, which are expected to lower cAMP levels [37], were also advantageous. Additionally, synthesis of the large cAMP-CRP regulon, which is expected to occur when the glucose in the media is exhausted [38], may not be the optimal allocation of cellular resources during antibiotic challenge. *Salmonella* strains lacking either *cyaA* or *crp* are more resistant to a wide range of antibiotics [39], suggesting that similar phenomena may occur with other organisms and antibiotics.

Other beneficial disruptions seem to alter the timing and magnitude of the stringent response, a program *E. coli* uses to redirect energy from rRNA and tRNA transcription to the creation of amino acid biosynthesis enzymes in response to amino acid starvation [40]. To accomplish the transition, Lon protease bound to polyphosphate degrades ribosomal proteins, freeing amino acids that can be incorporated into the needed enzymes [41], and guanosine tetraphosphate (ppGpp) and guanosine pentaphosphate (pppGpp) bind RNA polymerase, altering promoter selectivity [42]. Transposon insertions near *gpp* or *spoT*, genes whose products affect the levels and ratio of ppGpp and pppGpp [43], are beneficial in aminoglycosides (Figure 4 and Table S5). Similarly, insertions near *ppk*, which encodes polyphosphate kinase [44], or *lon* are beneficial in tetracyclines (Table S6).

The advantageous character of *cpxA* and *cpxP* disruptions points to a role for the Cpx system, which helps *E. coli* respond to extracytoplasmic stress [45], in aminoglycoside susceptibility. At the core of the Cpx system are the CpxA histidine kinase, its cognate response regulator, CpxR, and CpxP, a periplasmic repressor of CpxA [45]. Recent work indicates that the presence of the wild-type Cpx system increases the number of hydroxyl radicals, the final output of the Fenton reaction death pathway, possibly through crosstalk with the Arc system [34]. Somewhat surprisingly, however, in the absence of CpxA, the cellular pool of CpxR is partially phosphorylated, and the Cpx pathway is active, not off [46]. Furthermore, some mutations that constitutively activate the Cpx system cause pleiotrophic effects including amikacin [47] and kanamycin resistance [48], possibly through the increased expression of drug efflux pumps [49]. Different levels of Cpx-Arc crosstalk between the various mutant and wild-type strains may account for these observations.

### Mutations Affecting Susceptibility to β-lactams

Excluding loci with a general effect on antibiotic tolerance, disruption of 33 loci was beneficial in all three β-lactams tested, while disruption of 12 loci was deleterious (Figure 5A). Many of the beneficial disruptions turn on the Rcs signaling pathway (Figure 5B), which Laubacher and Ades [50] previously found to contribute to β-lactam tolerance. Laubacher and Ades also showed that the tolerance is not dependent on the Rcs system's role in increasing capsule synthesis [50]; they did not, however, evaluate the importance of the system's function in suppressing flagella-based motility [51]. Since many other disruptions beneficial in β-lactams are in genes whose products participate in flagella assembly, we hypothesize that suppression of flagella synthesis is responsible for the beneficial effect of Rcs system activation.

To confirm that the lack of flagella confers an advantage in β-lactams, we focused on a *fliN::kan* mutant in the presence of ampicillin. In direct competition with the parental strain, the mutant has a small advantage in media without drug, likely due to the high energetic cost of motility [52] (Figure 2). With ampicillin, however, the advantage is much larger (Figure 2), possibly because energy is a more valuable commodity in antibiotic-stressed cells. Alternatively, since assembling a flagellum requires peptidoglycan hydrolysis [53] and β-lactams inhibit peptidoglycan transpeptidation, the combination of stresses may interact synergistically. Interestingly, the MIC of the *filN::kan* mutant as well as that of several other strains with increased fitness in ampicillin is within measurement error of that of the wild-type strain (data not shown), highlighting the ability of the selection method employed to identify mutations of small effect.

Disruptions expected to work synergistically with β-lactams to disrupt peptidoglycan integrity are particularly harmful (Figure 5A). For example, loss of either *mltB* or *slt*, which encode membrane-bound lytic murein transglycosylases [54], is deleterious. Disruptions of *ampG*, which encodes a transporter involved in recycling murein [55], or of *ampC*, which encodes a β-lactamase resistance protein [55], are also detrimental.

### Disruption of *folX* or *folM* is Beneficial in Drugs that Inhibit Folate Biosynthesis

The only locus whose disruption was beneficial in both sulfamonomethoxine and trimethoprim, two drugs that inhibit key steps in folate metabolism (Figure 6), was *folM*, which encodes one of *E. coli*'s two dihydrofolate reductases (DHFRs). *E. coli*'s other DHFR, FolA, is inhibited by trimethoprim [56], and *folA* is essential in minimal media unless *thyA* is also knocked out and the media is supplemented with thymidine [57]. In contrast, *folM* deletion strains have no major growth defects, and trimethoprim does not inhibit FolM [58]. A *folM::kan* mutant has a 5-fold higher MIC in sulfamonomethoxine than the wild-type strain, but no detectable change in MIC in trimethoprim (Table S2). To assess the strain's fitness more sensitively, we subjected the *folM::kan* strain to direct competition with the wild-type strain and found that the *folM::kan* mutation is neutral without drug and beneficial in the presence of trimethoprim (Figure 2). That the effects in sulfamonomethoxine are stronger than those in trimethoprim is consistent with the original transposon enrichment experiments, which needed two and four days to find loci affecting susceptibility to sulfamonomethoxine and trimethoprim, respectively. The beneficial nature of *folM* deletions in sulfamonomethoxine and trimethoprim is surprising, as deleting *folM* would naively be expected to reduce the available amount of DHFR, making a bad situation worse. We hypothesize that *E. coli* may respond to a lack of FolM by increasing FolA levels, ameliorating the effects of sulfamonomethoxine and trimethoprim.

Another gene connected to folate biosynthesis whose disruption was beneficial during the trimethoprim enrichment is *folX*. Although the *folX* data slightly missed the significance thresholds for the sulfamonomethoxine enrichment, deleting *folX* gave a ∼2-fold increase in MIC in sulfamonomethoxine (Table S2). Like *folM*, a *folX* deletion did not change the MIC in trimethoprim, but the mutant strain did have a competitive advantage over the wild-type strain in trimethoprim (Figure 2). FolX catalyzes the conversion of 7,8-dihydroneopterin triphosphate to dihydromonapterin-triphosphate [59], which redirects 7,8-dihydroneopterin triphosphate away from the synthesis of tetrahydrofolate (Figure 6). Thus, *folX* mutations likely allow metabolic compensation [60] by increasing the flux of metabolites through the folate biosynthesis pathway. In fact, increased flux from enhanced p-aminobenzoate production is a common mechanism of sulfamonomethoxine resistance [61].

### Loci Conferring a General Increase or Decrease in Antibiotic Susceptibility

**Figure 7:**
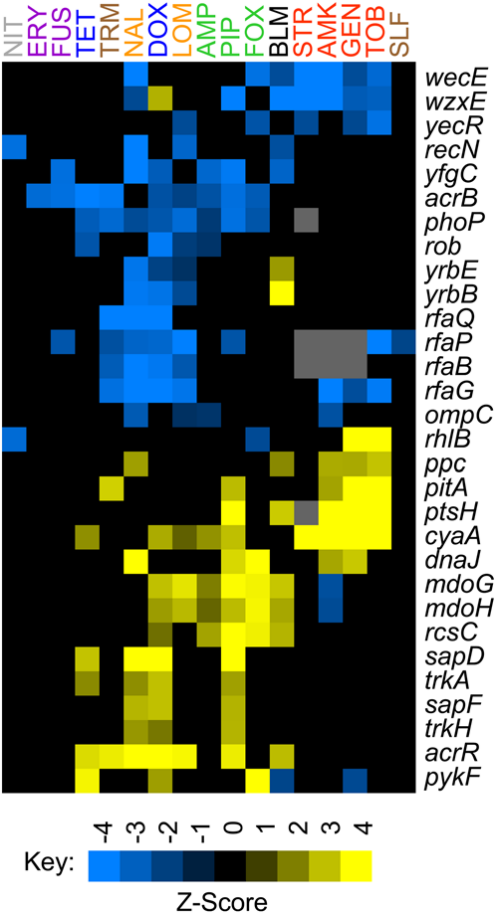
Genes altering susceptibility to three or more classes of antibiotics. Yellow (blue) indicates that transposon insertions in or near a gene were beneficial (deleterious). Black indicates no significant effect; gray indicates missing data. Antibiotics with the same target are written in the same color. Sulfamonomethoxine and trimethoprim inhibit different enzymes in the folic acid biosynthesis pathway; placing them in separate classes did not alter the results. Z-scores were calculated as described in Materials and Methods.

Transposon insertions in or near 30 genes provided a significant change in fitness in at least three antibiotics with distinct targets (Figure 7). While some drugs of the same class, such as the aminoglycosides have similar fitness profiles, a mutant's behavior in the presence of a drug cannot generally be determined based solely on knowledge of the drug's mechanism of action. This is especially true for drugs of the same class, such as tetracycline and doxycycline, which have distinct chemical properties that restrict them to different routes of entry into the cell. In particular, the comparatively hydrophilic tetracycline passes through OmpF porins while the more hydrophobic doxycycline diffuses through the outer membrane [62].

*E. coli* generates multiple barriers to protect itself from different classes of harmful, foreign compounds, and we found, as expected, that many of the loci responsible for general alterations in susceptibility encode enzymes that synthesize cell envelope components. In particular, perturbations to the negatively-charged lipopolysaccharides (LPS) and enterobacteria common antigen (ECA) that protect the outer membrane [63], [64] were widely deleterious. Defective LPS is known to increase sensitivity to hydrophobic antibiotics and polycationic compounds such as aminoglycosides [63]. ECA, on the other hand, is thought to provide protection against organic acids [65], but its role in antibiotic tolerance had not been well explored; Tamae et al. [20] did, however, report that loss of *rffA* increases susceptibility to gentamycin. Strains with defective ECA (i.e., *wzxE::kan*) and LPS (i.e., *rfaG::kan*) generally had lower MICs than wild-type in the same antibiotics in which they were depleted during the selections (Table S7). Direct competitions with a wild-type strain confirmed that a Δ*wzxE* mutation is neutral in media without drug and deleterious in both nalidixic acid and amikacin (Figure 2).

Rarely was the disruption of a locus beneficial in the presence of some drugs and deleterious in others. Notable exceptions are *yrbB* and *yrbE,* whose products belong to a system that prevents mislocalized phospholipids from accumulating in the outer membrane's outer leaflet [66]. We confirmed that a *yrbE::kan* strain has a higher MIC than the wildtype in bleomycin, has similar tolerance to the wildtype in tetracycline, and is more susceptible than the wildtype to nalidixic acid, lomefloxacin, and doxycycline (Table S7). Loss of the Yrb system likely has little effect in tetracycline because tetracycline enters the cell using porins rather than passing directly through the outer membrane [62]. Defects in the Yrb system likely reduce the negative charge on the outer membrane, which would decrease the permeability to positively charged bleomycin and increase the permeability towards the more neutral and negatively charged nalidixic acid, lomefloxacin, and doxycycline. The mechanism may be similar to how expression of the PmrA regulon, which makes the LPS less negative, increases *E. coli*'s tolerance to the positively charged polymyxin B but also increases susceptibility to anionic detergents [67].

To increase antibiotic tolerance, bacteria often reduce the intracellular drug concentration by increasing the expression of efflux pumps that use either ATP or membrane potential to expel toxic agents [68]. Not surprisingly, disruption of genes that control the levels of the AcrB/AcrA*/*TolC system, *E. coli*'s main drug efflux pump [69], alters tolerance to multiple antibiotics. Disruption of the *acrB* gene is widely deleterious (Figure 7). Disruptions of *phoP* or *rob*, which encode transcriptional activators of the *acrAB* operon [70], [71], are also deleterious, while disruption of *acrR*, which encodes a transcriptional repressor of the *acrAB* operon [72], is beneficial. Disruption of no other drug pump was generally deleterious.

Disruption of *E. coli*'s Trk potassium transport system was beneficial in a wide range of antibiotics. The low-affinity transporter contains TrkA, TrkE (SapD), and either TrkH or TrkG proteins [73], [74], and the Tn-enrichment experiment indicated that disruptions in *sapD*, *trkA*, and *trkH* are beneficial in piperacillin, doxycycline, tetracycline, and nalidixic acid (Figure 7). Work with a *sapD::kan* mutant indicated that removal of the system increases the MIC by about 1.5-fold in doxycycline, tetracycline, and nalidixic acid (Table S7). In direct competitions between a Δ*sapD* mutant and the parental strain, the Δ*sapD* mutation was slightly deleterious in the absence of drug, beneficial in the presence of nalidixic acid, and neutral with tetracycline (Figure 2). The connection between potassium transport and antibiotic tolerance merits further study.

Notably, disruptions of two genes of unknown function, *yecR* and *yfgC*, are deleterious (Figure 7). Tamae et al. [20] found that removal of *yfgC* decreases the MIC in vancomycin, rifampicin, and ampicillin. We confirmed the result for ampicillin, and we found that the MIC is also lower than wild-type in fusidic acid, doxycycline, and trimethoprim (Table S7). *yfgC* has homology to peptidases, and PSORTb [75] predicts that the protein is in the inner membrane. *yecR* is regulated by FlhDC [76] and has homology to lipoproteins. Strong homologs to *yecR* are found only in other *Enterobacter* species; homologs for *yfgC* are slightly more widespread, appearing in multiple gamma-proteobacteria.

### Resistance through Accumulation of Mutations of Small Effect

To explore the potential for *E. coli* to acquire higher levels of antibiotic tolerance through the sequential accumulation of the identified chromosomal mutations, we constructed several double mutants. For each of two drugs, from among the genes that gave measurable MIC increases when removed singly, we chose pairs that were putatively in different pathways. As expected, the double mutants exhibited higher MICs than the parental strain and both of the two single mutant derivatives. In particular, *ybjC::kan* and *ompR::kan* mutants have MICs 2.25-fold greater than the wild-type strain in nitrofurantoin, while a Δ*ompR ybjC::kan* double mutant has a MIC 5-fold greater than the wild-type strain. The *ybjC::kan* allele's beneficial effects likely come from reduced expression of the downstream *nfsA* gene, which encodes an oxygen-insensitive nitroreductase that converts nitrofurantoin into toxic intermediates [77]. Similarly, MICs of *sapD::kan* and *lon::kan* mutants in tetracycline are 1.5-fold higher than the wild-type strain, and the MIC of a Δ*sapD lon::kan* mutant is 2.25-fold greater than the wild-type parent.

A wide variety of mutations, including single base pair changes, can cause the null phenotypes attained in this work through transposon insertions and gene replacements. Furthermore, for most antibiotics, the set of beneficial disruptions spans multiple pathways. Thus, *E. coli*'s current genome is likely mutationally close to one conferring significantly higher antibiotic tolerance. A clinical *S. aureus* strain was observed acquiring 35 chromosomal mutations on the way to vancomycin resistance [78], and in the laboratory, multiple weak chromosomal mutations have been combined to give higher resistance in both *P. aeruginosa* [24] and *Helicobacter pylori* [23], suggesting that the phenomenon is quite general.

### Concluding Remarks

In this work, we competitively grew transposon insertion mutants of *E. coli* in batch cultures with drug concentrations that had a moderate impact on the parental strain's growth rate. Propagating the mutant collection for a sufficiently long duration allowed us to identify both beneficial and deleterious mutations of a wide range of strengths. Our analyses reveal that *E. coli* has a large mutational target size for altering its antibiotic tolerance.

As the disruption of the genes identified in this study pushes cells from the growth regime of moderate inhibition towards one of the extremes of no inhibition or full inhibition, the products of the loci and the pathways in which they reside are promising starting points for the development of adjuvant therapies. For example, if a gene's deletion causes hypersensitivity to an antibiotic, that antibiotic and a drug targeting the corresponding gene product may act synergistically. Similarly, when disrupting a pathway increases bacterial fitness in the presence of a particular antibiotic, stimulating the pathway might enhance the antibiotic's efficacy. The development of such adjuvant therapies has the potential to expand the usefulness of the limited set of antibiotics currently available.

With whole-genome sequencing becoming increasingly affordable, this work should provide a wealth of data for interpreting mutations present in drug resistant, pathogenic strains. As approximately half of the genes identified as altering fitness in the presence of antibiotics increase tolerance when disrupted, it will be important to learn how frequently and in what combinations the adaptive building blocks revealed here appear in clinical and environmental settings. The bulk of the loci identified occur in multiple species, and future work will be needed to discover how specific the beneficial and deleterious nature of each perturbation is to the wiring of *E. coli*'s cellular network. Our observations should provide a scaffold for understanding the contribution of chromosomal mutations to antibiotic resistance as well as an aid in the development of novel therapeutics.

## Materials and Methods

### Bacterial Strains and Growth Conditions

All experiments were performed using *E. coli* MG1655 [79]. Transposon insertion mutants were generated in a MG1655 Δ*lacZ* strain as described in a previous study [25]. All experiments were conducted in M9 salts [80] supplemented with 0.4% glucose, 0.1% casamino acids, 1 mM MgSO_4_, 0.1 mM CaCl_2_, and 1.5 μM thiamine. LB media contained 0.1% Bacto Tryptone, 0.05% yeast extract, and 0.05% NaCl. All antibiotics were purchased from Sigma. Unless otherwise noted, cultures were shaken at 37°C.

### Transposon Library Enrichments, DNA manipulation, and Hybridization

Genetic footprinting and subsequent hybridization to DNA spotted arrays were performed as described in Girgis et al. [25]. As a starting point, we hybridized DNA from the day in which minor banding patterns began to emerge (Figure 1C) and then adjusted the chosen day as necessary. On early days during a selection, a mutant's fitness did not have a measurable effect on its prevalence, while on very late days, only a few types of mutants remained, and the relative fitness of the mutants that completely dropped out could not be discerned. In a few cases (Table 1), data from adjacent days of roughly equal suitability were included in the analysis. Other hybridizations from days not ultimately chosen exhibited either extreme selection or little to no selection and were excluded. The distinct behavior of the library in each antibiotic necessitated the choice of different days for different antibiotics (Table 1). To reduce the chance of spontaneous mutations overtaking the cultures, to remove the need for additional sets of controls for comparison, and to focus on transposon insertions causing larger effects, no samples from days 5–7 were chosen. Samples from at least two independent replicate selections were hybridized for each antibiotic. As controls, six samples from independent selections in the absence of any drug were hybridized.

### Determining Significant Changes

Ratios (transposon signal/genomic DNA signal) from the antibiotic enrichments were compared to both the ratios from the original unselected library and to ratios from enrichments of the transposon library performed in identical media without antibiotics. Two z-scores were calculated for each ratio, r, where z = (x−μ)/σ, x = log_2_(r), and μ and σ are the mean and standard deviation, respectively, of the log_2_ ratios for the gene from reference hybridizations. One z-score used five reference hybridizations of the unselected library (from Girgis et al. [25]) and the other used six reference hybridizations of the library selected in the same media without antibiotics. All six no-antibiotic samples came from independent selections; three selections lasted two days, and three lasted four days.

To identify the most reproducible fitness effects, we considered all of the z-scores for each gene for a given antibiotic. (Antibiotics with two and three hybridizations had four and six z-scores, respectively.) When all of the z-scores had the same sign, we assigned the gene the z-score in the set that was closest to zero (representing the smallest effect). When a gene had z-scores of different signs, the gene was assigned a score of 0, indicating no consistent fitness effect. Supplementary information contains normalized ratios (Dataset S2), z-scores relative to the unselected library (Dataset S3), z-scores relative to the enrichments performed in the media without antibiotics (Dataset S4), combined z-scores (Dataset S5), and the combined z-scores considered significant (Dataset S1).

The significance threshold was set so that two false positives are expected per antibiotic. False positives were estimated by treating randomly chosen reference samples as data and repeating the analysis procedure. (See Text S1.)

### Determining Genes Common to Aminoglycosides and β-lactams

In identifying loci important to fitness in specific antibiotic classes, care was taken to prevent the exclusion of genes that barely missed the significance cutoff for a subset of the drugs. As such, a locus was considered to be beneficial or deleterious in aminoglycosides if *i*) the prevalence of mutants where the locus was disrupted changed significantly during the enrichments for at least two of the four drugs and *ii*) the z-scores for the locus for all four drugs had the same sign. For example, disruption of a locus was classified as beneficial in aminoglycosides if the gene had positive z-scores in all four drugs, and the z-scores reached the significance level for at least two drugs. β-lactams were treated similarly except that the disruption of a locus was required to cause a significant fitness change during the enrichments for at least one of three drugs. Loci with a general effect on antibiotic tolerance were excluded from the sets.

### Strain Construction

Rather than choosing one of the many mutants in the library with a transposon inserted in a particular gene, we corroborated behavior observed during the selections using strains where the gene of interest had either been replaced with a kanamycin (*kan*) resistance cassette or removed to create an in-frame deletion. To construct the strains, *P1vir* transduction [81] was used to move the necessary alleles from the Keio collection [82] to MG1655 [79]. To create unmarked, in-frame deletions, the *kan* cassette was removed using FLP recombinase [83]. In rare cases, both the original transposon insertions as well as the kanamycin resistance cassette can produce polar effects, resulting in mutants with phenotypes distinct from the null phenotype of the disrupted or replaced gene.

### Software Used

Data was clustered with Cluster [84] and visualized using Treeview [84]. Data manipulations were performed using Perl and Matlab. iPAGE (Hani Goodarzi, unpublished data) was used to examine sets of genes for enrichments in GO category, transcription factor regulon, and stress response membership. Annotations came from EcoCyc [85] and genome-tools [86].

## Supporting Information

Text S1 Additional Materials and Methods
(0.07 MB PDF)

Figure S1 Dose Response Curves Used to Select Drug Concentrations. For each antibiotic, fresh media containing various drug concentrations was inoculated with overnight culture of the wild-type strain. Cultures were shaken at 37°C, and growth was monitored using OD600 readings. Blue indicates the concentrations chosen for the enrichments.
(0.18 MB PDF)

Figure S2 Gel images from enrichments done in the study media in the absence of antibiotics. Shown are the amplified Tn-adjacent DNA from all seven days for each of the seven repetitions. DNA was amplified as described in Girgis et al. [1] and separated on a 2% agarose gel. Yellow rectangles indicate samples hybridized. From the bottom, marker sizes are 100, 200, 300, 400, 500, 650, 850, and 1000 bases.
(1.48 MB PDF)

Figure S3 Gel images from Tn-insertion library enrichments done in the presence of antibiotics. Shown are the amplified Tn-adjacent DNA from all seven days for each of the three repetitions done for each antibiotic. DNA was amplified as described in Girgis et al. [1] and separated on a 2% agarose gel. Yellow rectangles indicate samples hybridized. From the bottom, marker sizes are 100, 200, 300, 400, 500, 650, 850, and 1000 bases.
(2.10 MB PDF)

Figure S4 Loci whose disruption was significant in at least one quinolone. Yellow (blue) indicates that transposon insertions in or near a gene were beneficial (deleterious). Black indicates no significant effect. Z-scores were calculated as described in Materials and Methods.
(0.21 MB PDF)

Figure S5 Loci whose disruption was significant in at least one tetracycline. Yellow (blue) indicates that transposon insertions in or near a gene were beneficial (deleterious). Black indicates no significant effect; gray indicates missing data. Z-scores were calculated as described in Materials and Methods.
(0.24 MB PDF)

Figure S6 Loci whose disruption was significant in at least one folic acid biosynthesis inhibitor. Yellow (blue) indicates that transposon insertions in or near a gene were beneficial (deleterious). Black indicates no significant effect; gray indicates missing data. Z-scores were calculated as described in Materials and Methods.
(0.13 MB PDF)

Figure S7 Loci whose disruption was significant in at least one inhibitor of the 50S subunit of the ribosome. Yellow (blue) indicates that transposon insertions in or near a gene were beneficial (deleterious). Black indicates no significant effect. Z-scores were calculated as described in Materials and Methods.
(0.08 MB PDF)

Figure S8 Loci whose disruption was significant in bleomycin. Yellow (blue) indicates that transposon insertions in or near a gene were beneficial (deleterious). Z-scores were calculated as described in Materials and Methods.
(0.13 MB PDF)

Figure S9 Loci whose disruption was significant in at least one β-lactam. Yellow (blue) indicates that transposon insertions in or near a gene were beneficial (deleterious). Black indicates no significant effect. Z-scores were calculated as described in Materials and Methods. Note that this set of loci is distinct from the set of loci whose disruption caused significant changes in all the beta-lactams tested (Table S2).
(0.10 MB PDF)

Figure S10 Loci whose disruption was significant in nitrofurantoin. Yellow (blue) indicates that transposon insertions in or near a gene were beneficial (deleterious). Z-scores were calculated as described in Methods.
(0.09 MB PDF)

Figure S11 Loci whose disruption was significant in at least one aminoglycoside. Due to the large size of the set, genes whose disruption was only significant in tobramycin are not shown. Data for tobramycin is available in Dataset S1. Yellow (blue) indicates that transposon insertions in or near a gene were beneficial (deleterious). Black indicates no significant effect; gray indicates missing data.
(0.24 MB PDF)

Table S1 Loci that changed susceptibility to all aminoglycosides tested.
(0.08 MB PDF)

Table S2 Loci that changed susceptibility to all beta-lactams tested.
(0.07 MB PDF)

Table S3 Genes identified in this work as having a general role in antibiotic susceptibility.
(0.07 MB PDF)

Table S4 Additional genes identified in both this study and previous work.
(0.07 MB PDF)

Table S5 MIC changes in aminoglycosides.
(0.06 MB PDF)

Table S6 Additional class-specific MIC changes (non-aminoglycosides).
(0.07 MB PDF)

Table S7 MIC changes for mutants with altered susceptibility to multiple drug classes.
(0.07 MB PDF)

Dataset S1 Z-scores for loci with a significant effect on antibiotic susceptibility.
(0.90 MB XLS)

Dataset S2 Normalized ratios (transposon signal/genomic DNA signal)
(4.23 MB XLS)

Dataset S3 Z-scores for individual hybridization computed relative to five hybridizations of the original, unselected library.
(3.58 MB XLS)

Dataset S4 Z-scores for individual hybridizations computed relative to six hybridization of the library cultured in the same media (M9 with glucose and casamino acids) without antibiotics.
(3.58 MB XLS)

Dataset S5 Combined z-scores for all loci.
(1.24 MB XLS)
