# Supplementary material for: Genetic Architecture of Intrinsic Antibiotic Susceptibility
Source: PLoS One. 2009 May 20;4(5):e5629. doi: 10.1371/journal.pone.0005629 (PMC2680486; doi:10.1371/journal.pone.0005629)
Supplement: Text S1 — Additional Materials and Methods (0.07 MB PDF) [file pone.0005629.s001.pdf]

Text S1: Additional Materials and Methods

Array pre-processing

Array images were processed using GENEPIX PRO software versions 5.0 and 6.0. For each spot, the signal for each channel, *i* (genomic DNA or transposon library), was the spot's mean foreground intensity (F_i) minus the median of the local background intensity (B_i). If the genomic DNA channel's signal (F_{DNA}-B_{DNA}) was less than 10, the spot was excluded from further analysis as the denominator was too weak to allow accurate calculation of the ratio between the transposon signal and the genomic DNA signal. Negative transposon channel signals were set to zero. Abnormally small spots (diameter less than 100 pixels) were excluded. Signals from each channel were normalized by the array's total signal for that channel. Finally, the ratio for each spot was calculated as the normalized transposon signal divided by the normalized genomic DNA signal. Ratios from the duplicate spots on each slide were averaged. To facilitate between array comparisons, arrays were normalized so that the sum of the ratios for the set of genes present on all arrays (3334 genes) was 5000 (arbitrarily chosen).

For pipercillin, cefoxitin, tetracycline, and trimethoprim, two separate hybridizations were done for one of the samples. The data from the two arrays was similar. Corresponding values were averaged and treated as a single repetition during subsequent analysis.

Detailed Information on Z-Score Calculation

Ratios (transposon signal/genomic DNA signal) from the antibiotic enrichments were compared to both the ratios from the original unselected library and to ratios from enrichments of the transposon library done in identical media without antibiotics. Ratios smaller than 0.05 were set to 0.05. Two z-scores were calculated for each ratio, r, where $z = (x-\mu)/\sigma$, $x = \log_2(r)$, and μ and σ are the mean and standard deviation, respectively, of the $\log_2$ ratios for the gene from reference hybridizations. One z-score used five reference hybridizations of the unselected library (from [1]) and the other used six reference hybridizations of the library selected in the same media without antibiotics. All six no-antibiotic samples came from independent selections; three selections lasted two days, and three lasted four days.

To identify the most reproducible fitness effects, we considered all of the z-scores for each gene for a given antibiotic. (Antibiotics with two and three hybridizations had four and six z-scores, respectively.) When all of the z-scores had the same sign, we assigned the gene the z-score in the set that was closest to zero (representing the smallest effect). When a gene had z-scores of different signs, the gene was assigned a score of 0, indicating no consistent fitness effect. For a gene to be assigned a non-zero z-score, data from at least two repetitions was needed.

The above analysis assumes that the prevalence of the mutants with fitness equal to wild-type does not change appreciably in the course of the experiment. During some of the antibiotic selections, however, the prevalence of mutants with beneficial transposon insertions increased sufficiently to noticeably decrease the abundance of the "average" mutant. Furthermore, due to the relatively small number of reference samples employed, some of the standard deviations are artificially small. To reduce the sensitivity of the analysis to the standard deviations of individual genes and to the different distributions of the reference and experimental samples, a "global" component was added to all standard deviations. The value of 0.15 was chosen heuristically based on simulations of the number of false positives expected at significance thresholds that allowed the correct classification of loci known to be affected by the experimental perturbation. Smaller values gave unacceptably high false positive rates. The addition of a global component has the effect of reducing both the importance of the standard deviation of the reference sample and the chance that small differences between the means of the experimental and reference samples caused by shifting distributions will be erroneously judged significant.

Details on False Positive Rate Determination

The significance threshold was set so that two false positives are expected per antibiotic. False positives were estimated by treating randomly chosen reference samples as data and repeating the analysis procedure. For the basic procedure, for each gene, one of the six no-antibiotic selection samples and one of the five unselected library samples were randomly chosen. Then, a z-score was calculated as detailed above using the two chosen points as the data and remaining points as the two reference sets. The same procedure was repeated for 30 choices of random paired samples for each gene (119039 samples). Based on the distribution, a cutoff of 2.15 (positive or negative) was chosen as the threshold for antibiotics for which we had two hybridizations. For those antibiotics with three hybridizations, the same technique was used except that two samples from one reference set

and one from the other were designated as data, giving a cutoff of 1.5 for two false positives per antibiotic.

MIC Determination

MICs were determined in Costar 96-well flat bottom plates. Overnight M9-grown cultures were diluted 2000-fold into fresh M9 media containing 1.5-fold dilution series of antibiotics. After incubating the plates at 37°C for 24 hours, the lowest concentration of drug whose well did not contain visible growth was considered to be the MIC. Most strains were tested only with the drug with which they were expected to show differential behavior compared to wild-type. However, in cases where mutants were expected to behave differently with different drugs of the same class, such as doxycycline and tetracycline, MICs were determined for all members of the class. Values shown are the median of at least two repetitions. To avoid suggesting an inflated degree of precision, when an even number of samples were used and the two middle values differed, their average was conservatively rounded to the factor of 1.5-fold showing the smallest change from wild-type. In addition to Tables S1-S3, an additional ~30 strains were tested; most did not show measurable, reproducible changes and were not pursued further (data not shown).

Direct Fitness Competitions

LB-grown overnight cultures of the indicated mutants, in an MG1655 *lacZ*$^+$ background, and the library's parental strain (MG1655 *ΔlacZ*), were pelleted and washed with M9 media. Then, the cultures were mixed at 1:100 dilutions each into fresh M9. Cultures were shaken at 37°C, and every day 2% of the cultures were diluted into fresh media. Antibiotics were used at the concentration shown in Table 1. The numbers of each strain were monitored daily by plating appropriate dilutions on MacConkey indicator plates which allowed the red *lacZ*$^+$ and white *lacZ*$^-$ strains to be distinguished [2].

References

1. Girgis HS, Liu Y, Ryu WS, Tavazoie S (2007) A comprehensive genetic characterization of bacterial motility. PLoS Genet 3: 1644-1660.
2. MacConkey A (1905) Lactose-fermenting bacteria in faeces. J Hyg 5: 333-379.
