# Supplementary material for: Genetic Architecture of Intrinsic Antibiotic Susceptibility
Source: PLoS One. 2009 May 20;4(5):e5629. doi: 10.1371/journal.pone.0005629 (PMC2680486; doi:10.1371/journal.pone.0005629)
Supplement: Figure S1 — Dose Response Curves Used to Select Drug Concentrations. For each antibiotic, fresh media containing various drug concentrations was inoculated with overnight culture of the wild-type strain. Cultures were shaken at 37°C, and growth was monitored using OD600 readings. Blue indicates the concentrations chosen for the enrichments. (0.18 MB PDF) [file pone.0005629.s002.pdf]

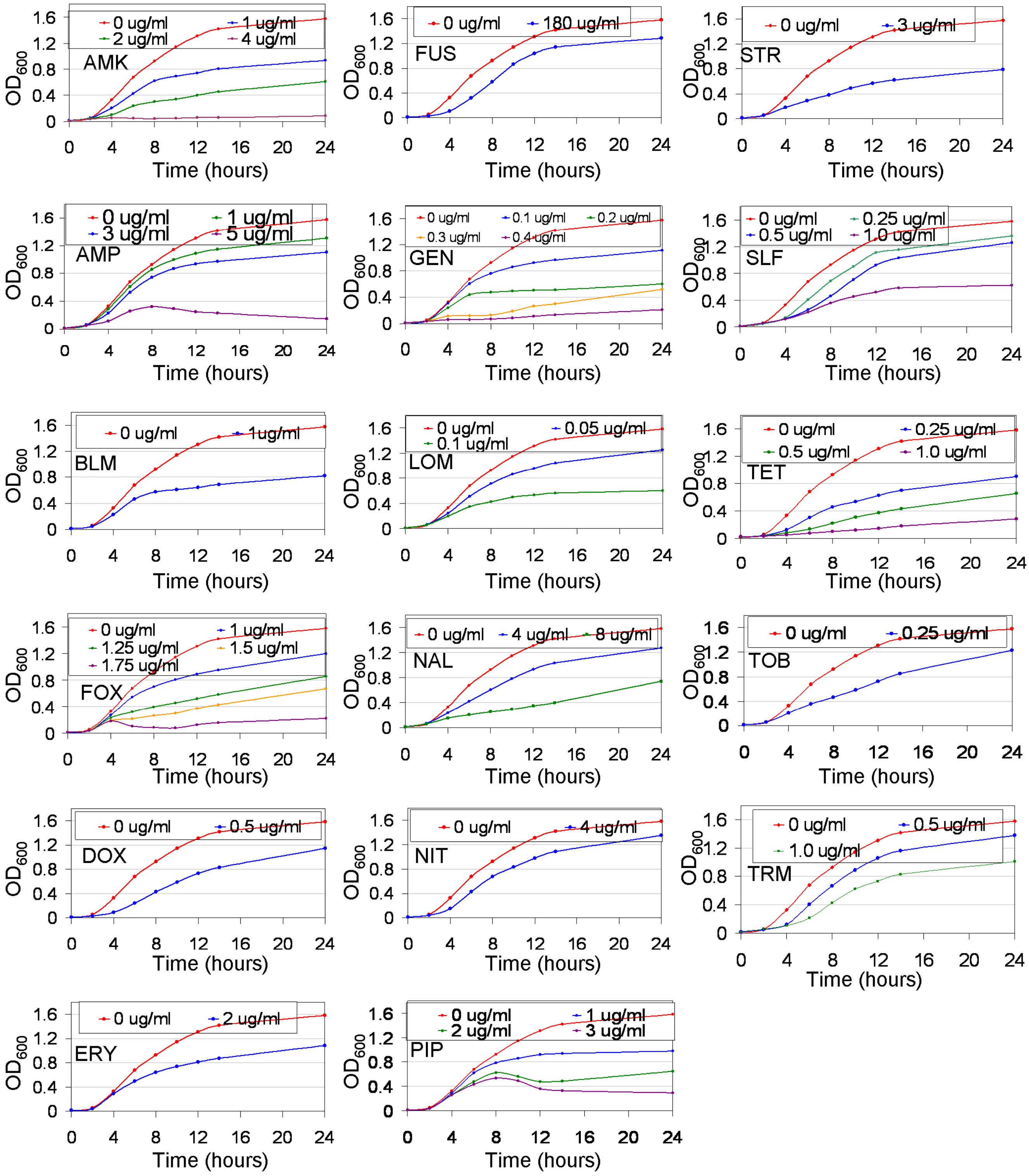

Figure S1. Dose Response Curves Used to Select Drug Concentrations.
For each antibiotic, fresh media containing various drug concentrations was inoculated with overnight culture of the wild-type strain. Cultures were shaken at 37°C, and growth was monitored using OD_{600} readings. Blue indicates the concentrations chosen for the enrichments.
