# Supplementary material for: Genetic Architecture of Intrinsic Antibiotic Susceptibility
Source: PLoS One. 2009 May 20;4(5):e5629. doi: 10.1371/journal.pone.0005629 (PMC2680486; doi:10.1371/journal.pone.0005629)
Supplement: Figure S2 — Gel images from enrichments done in the study media in the absence of antibiotics. Shown are the amplified Tn-adjacent DNA from all seven days for each of the seven repetitions. DNA was amplified as described in Girgis et al. [1] and separated on a 2% agarose gel. Yellow rectangles indicate samples hybridized. From the bottom, marker sizes are 100, 200, 300, 400, 500, 650, 850, and 1000 bases. (1.48 MB PDF) [file pone.0005629.s003.pdf]

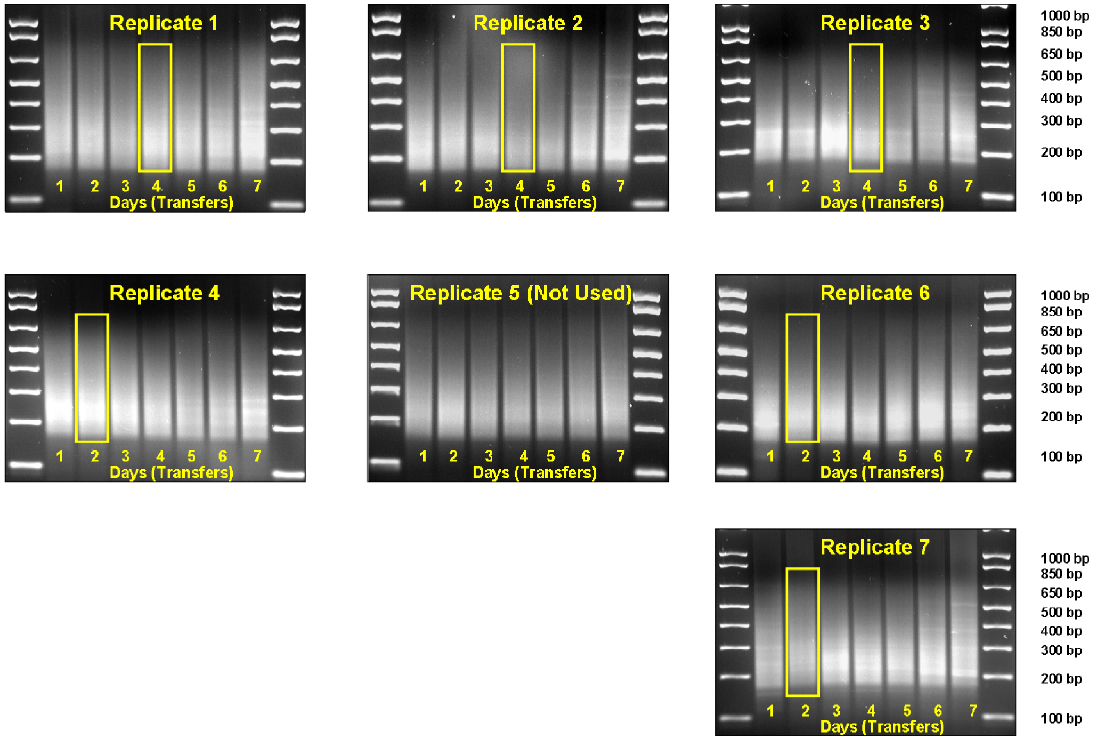

Figure S2. Gel images from enrichments done in the study media in the absence of antibiotics. Shown are the amplified Tn-adjacent DNA from all seven days for each of the seven repetitions. DNA was amplified as described in Girgis et al. [1] and separated on a 2% agarose gel. Yellow rectangles indicate samples hybridized. From the bottom, marker sizes are 100, 200, 300, 400, 500, 650, 850, and 1000 bases.

1. Girgis HS, Liu Y, Ryu WS, Tavazoie S (2007) A comprehensive genetic characterization of bacterial motility. PLoS Genet 3: 1644-1660.
