# Supplementary material for: Genetic Architecture of Intrinsic Antibiotic Susceptibility
Source: PLoS One. 2009 May 20;4(5):e5629. doi: 10.1371/journal.pone.0005629 (PMC2680486; doi:10.1371/journal.pone.0005629)
Supplement: Figure S3 — Gel images from Tn-insertion library enrichments done in the presence of antibiotics. Shown are the amplified Tn-adjacent DNA from all seven days for each of the three repetitions done for each antibiotic. DNA was amplified as described in Girgis et al. [1] and separated on a 2% agarose gel. Yellow rectangles indicate samples hybridized. From the bottom, marker sizes are 100, 200, 300, 400, 500, 650, 850, and 1000 bases. (2.10 MB PDF) [file pone.0005629.s004.pdf]

Figure 3 Part 1

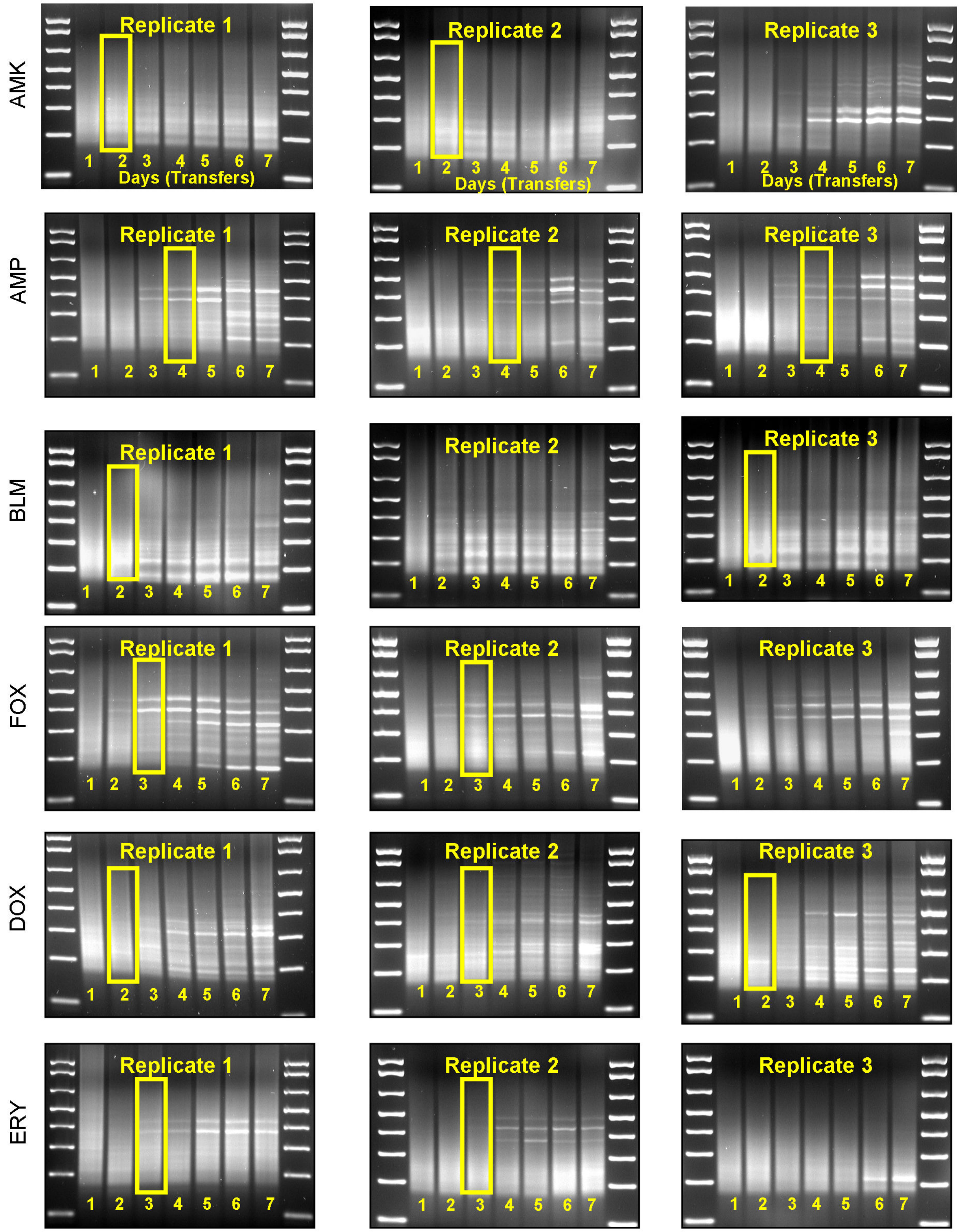

Figure 3 Part 2

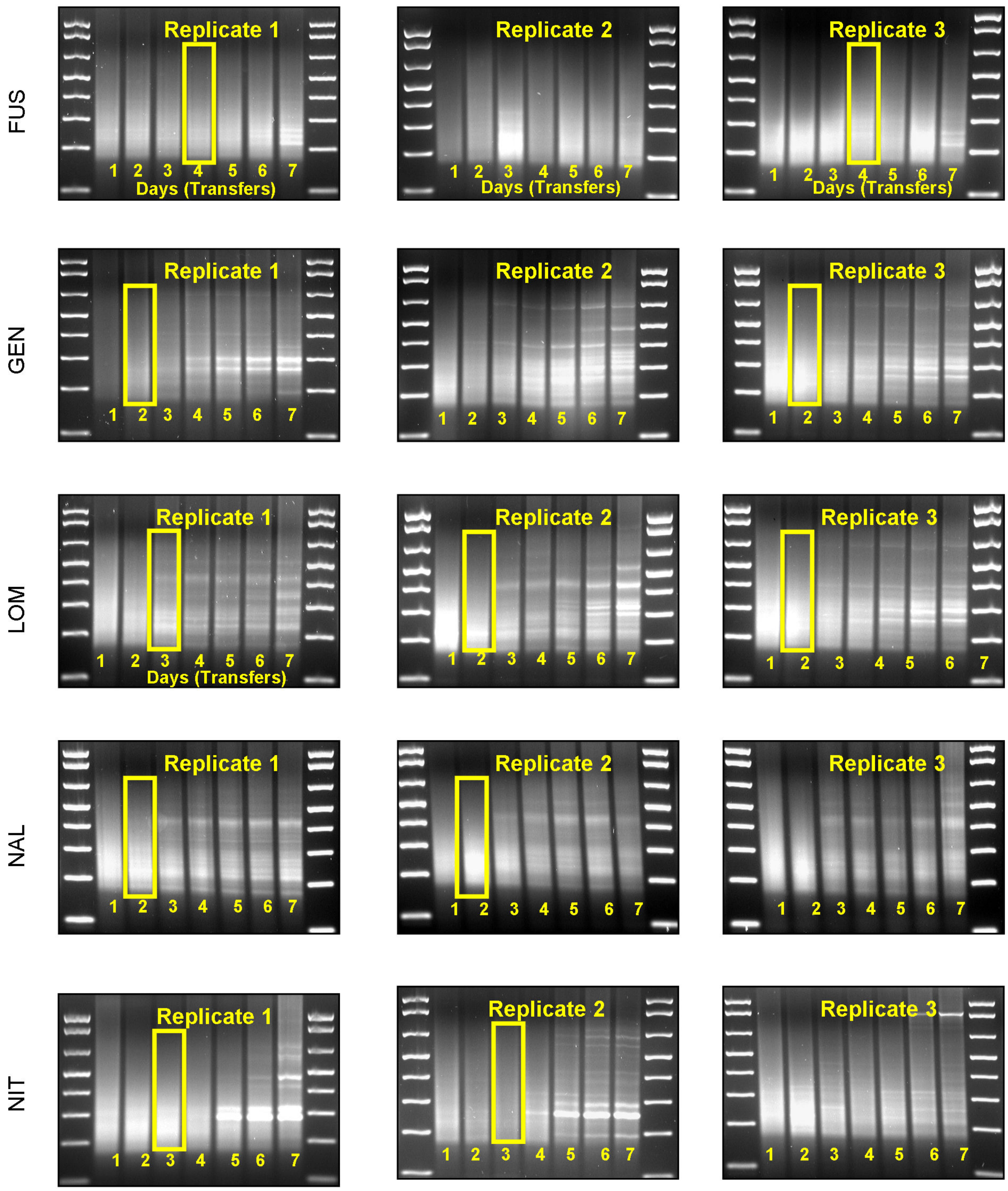

Figure 3 Part 3

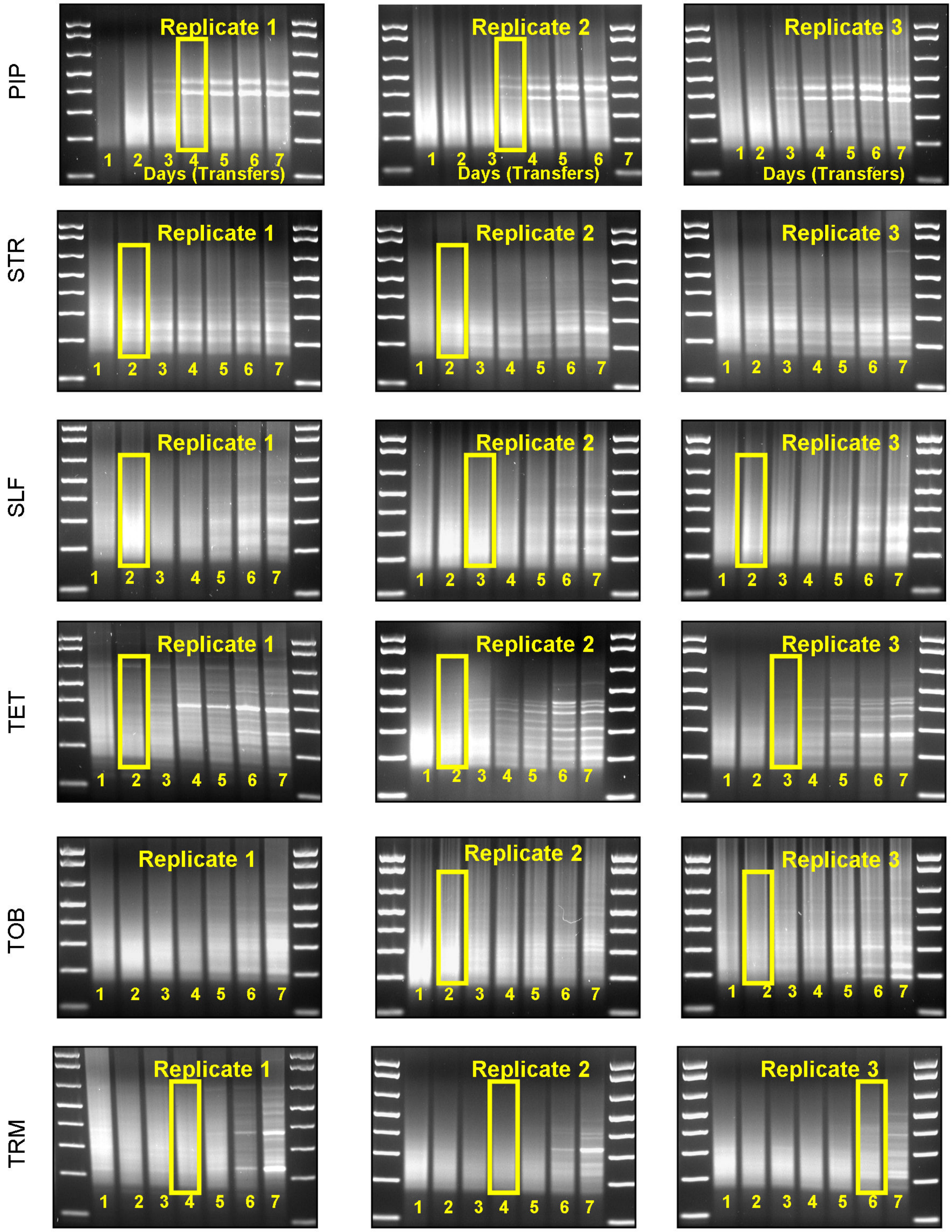

Figure S3. **Gel images from Tn-insertion library enrichments done in the presence of antibiotics.** Shown are the amplified Tn-adjacent DNA from all seven days for each of the three repetitions done for each antibiotic. DNA was amplified as described in Girgis et al. [1] and separated on a 2% agarose gel. Yellow rectangles indicate samples hybridized. From the bottom, marker sizes are 100, 200, 300, 400, 500, 650, 850, and 1000 bases.

1. Girgis HS, Liu Y, Ryu WS, Tavazoie S (2007) A comprehensive genetic characterization of bacterial motility. PLoS Genet 3: 1644-1660.
