# Supplementary material for: Genetic Architecture of Intrinsic Antibiotic Susceptibility
Source: PLoS One. 2009 May 20;4(5):e5629. doi: 10.1371/journal.pone.0005629 (PMC2680486; doi:10.1371/journal.pone.0005629)
Supplement: Figure S4 — Loci whose disruption was significant in at least one quinolone. Yellow (blue) indicates that transposon insertions in or near a gene were beneficial (deleterious). Black indicates no significant effect. Z-scores were calculated as described in Materials and Methods. (0.21 MB PDF) [file pone.0005629.s005.pdf]

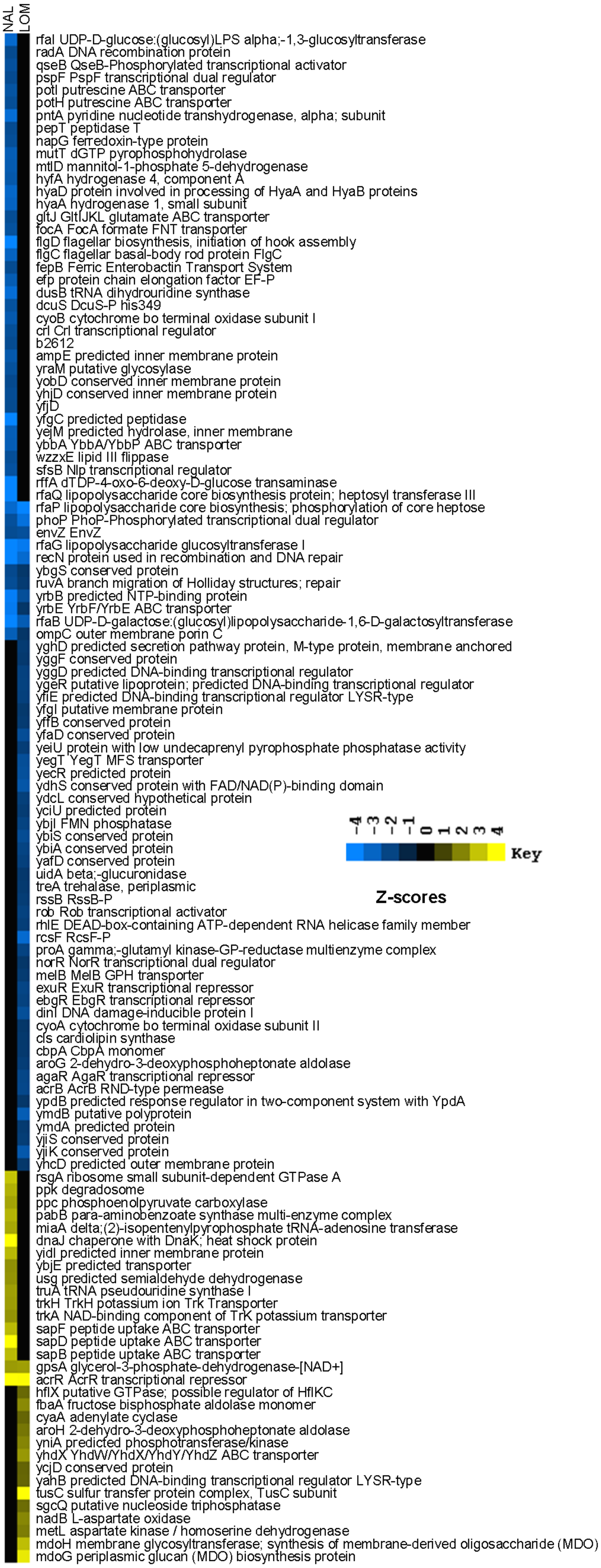

Figure S4. Loci whose disruption was significant in at least one quinolone.
Yellow (blue) indicates that transposon insertions in or near a gene were beneficial (deleterious). Black indicates no significant effect. Z-scores were calculated as described in *Materials and Methods*.
