# Supplementary material for: Genetic Architecture of Intrinsic Antibiotic Susceptibility
Source: PLoS One. 2009 May 20;4(5):e5629. doi: 10.1371/journal.pone.0005629 (PMC2680486; doi:10.1371/journal.pone.0005629)
Supplement: Figure S7 — Loci whose disruption was significant in at least one inhibitor of the 50S subunit of the ribosome. Yellow (blue) indicates that transposon insertions in or near a gene were beneficial (deleterious). Black indicates no significant effect. Z-scores were calculated as described in Materials and Methods. (0.08 MB PDF) [file pone.0005629.s008.pdf]

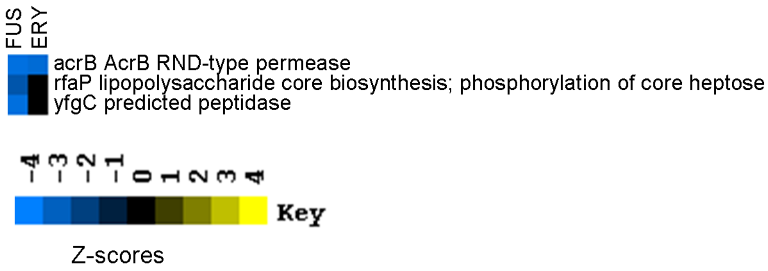

Figure S7. Loci whose disruption was significant in at least one inhibitor of the 50S subunit of the ribosome. Yellow (blue) indicates that transposon insertions in or near a gene were beneficial (deleterious). Black indicates no significant effect. Z-scores were calculated as described in *Materials and Methods*.
