# Supplementary material for: Genetic Architecture of Intrinsic Antibiotic Susceptibility
Source: PLoS One. 2009 May 20;4(5):e5629. doi: 10.1371/journal.pone.0005629 (PMC2680486; doi:10.1371/journal.pone.0005629)
Supplement: Figure S8 — Loci whose disruption was significant in bleomycin. Yellow (blue) indicates that transposon insertions in or near a gene were beneficial (deleterious). Z-scores were calculated as described in Materials and Methods. (0.13 MB PDF) [file pone.0005629.s009.pdf]

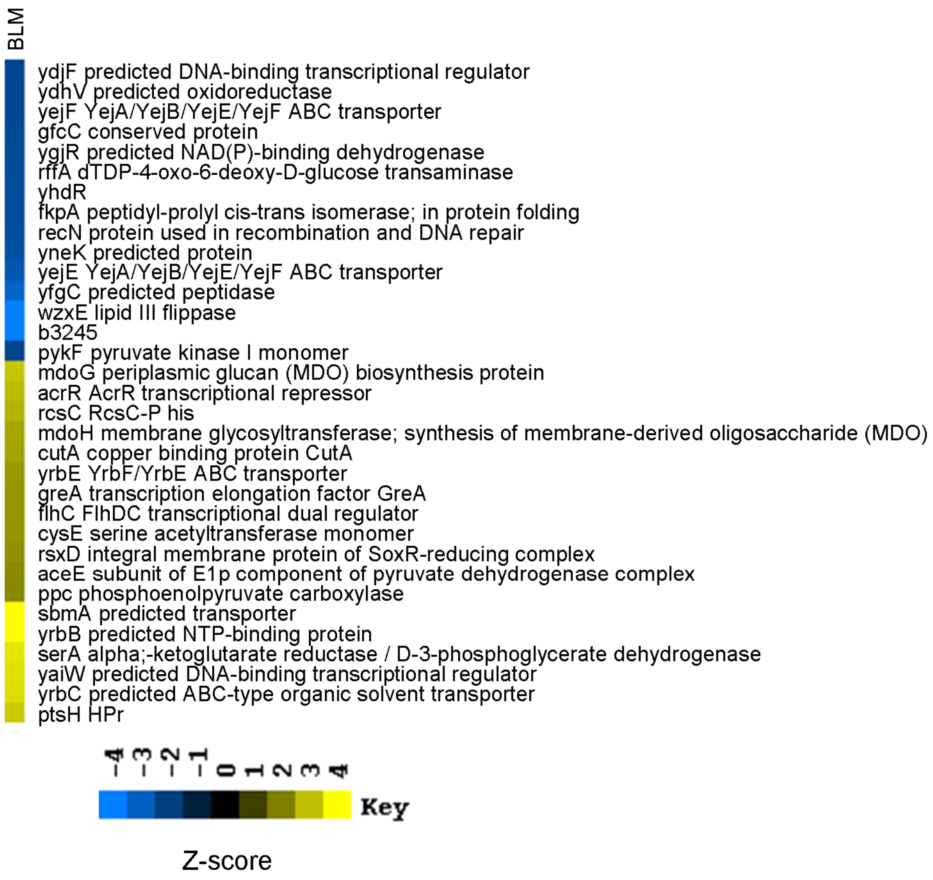

Figure S8. Loci whose disruption was significant in bleomycin.
Yellow (blue) indicates that transposon insertions in or near a gene were beneficial (deleterious). Z-scores were calculated as described in *Materials and Methods*.
