# Supplementary material for: Genetic Architecture of Intrinsic Antibiotic Susceptibility
Source: PLoS One. 2009 May 20;4(5):e5629. doi: 10.1371/journal.pone.0005629 (PMC2680486; doi:10.1371/journal.pone.0005629)
Supplement: Figure S9 — Loci whose disruption was significant in at least one β-lactam. Yellow (blue) indicates that transposon insertions in or near a gene were beneficial (deleterious). Black indicates no significant effect. Z-scores were calculated as described in Materials and Methods. Note that this set of loci is distinct from the set of loci whose disruption caused significant changes in all the beta-lactams tested (Table S2). (0.10 MB PDF) [file pone.0005629.s010.pdf]

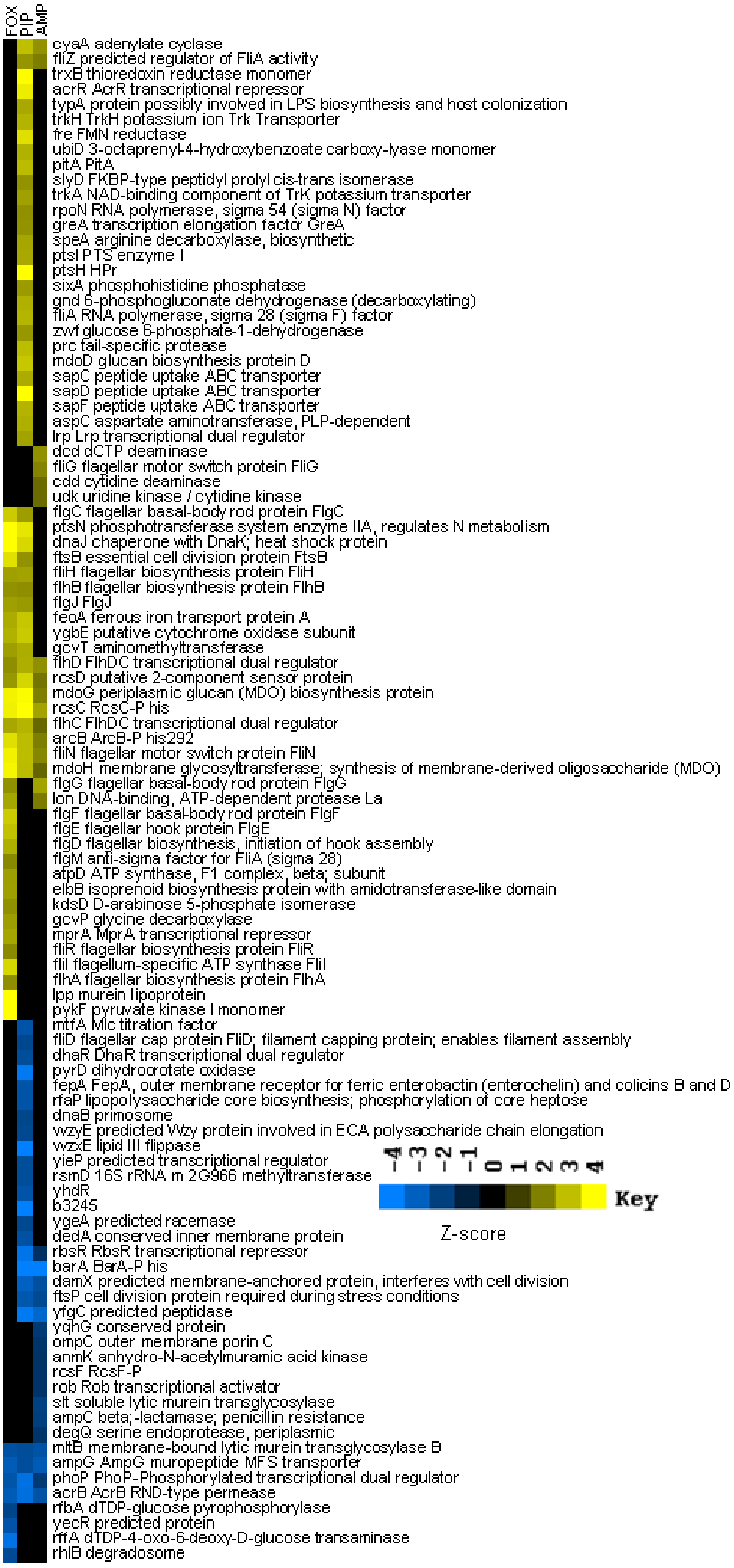

Figure S9. Loci whose disruption was significant in at least one β-lactam.
Yellow (blue) indicates that transposon insertions in or near a gene were beneficial (deleterious). Black indicates no significant effect. Z-scores were calculated as described in *Materials and Methods*. Note that this set of loci is distinct from the set of loci whose disruption caused significant changes in all the β-lactams tested (Table S2).
