# Supplementary material for: Genetic Architecture of Intrinsic Antibiotic Susceptibility
Source: PLoS One. 2009 May 20;4(5):e5629. doi: 10.1371/journal.pone.0005629 (PMC2680486; doi:10.1371/journal.pone.0005629)
Supplement: Figure S10 — Loci whose disruption was significant in nitrofurantoin. Yellow (blue) indicates that transposon insertions in or near a gene were beneficial (deleterious). Z-scores were calculated as described in Methods. (0.09 MB PDF) [file pone.0005629.s011.pdf]

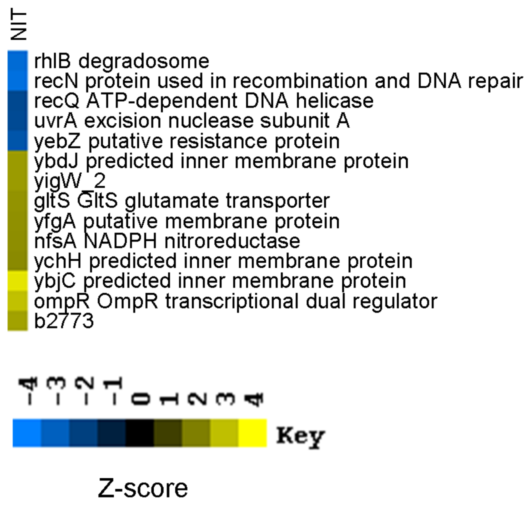

Figure S10. Loci whose disruption was significant in nitrofurantoin.
Yellow (blue) indicates that transposon insertions in or near a gene were beneficial (deleterious). Z-scores were calculated as described in *Methods*.
