# Supplementary material for: Genetic Architecture of Intrinsic Antibiotic Susceptibility
Source: PLoS One. 2009 May 20;4(5):e5629. doi: 10.1371/journal.pone.0005629 (PMC2680486; doi:10.1371/journal.pone.0005629)
Supplement: Figure S11 — Loci whose disruption was significant in at least one aminoglycoside. Due to the large size of the set, genes whose disruption was only significant in tobramycin are not shown. Data for tobramycin is available in Dataset S1. Yellow (blue) indicates that transposon insertions in or near a gene were beneficial (deleterious). Black indicates no significant effect; gray indicates missing data. (0.24 MB PDF) [file pone.0005629.s012.pdf]

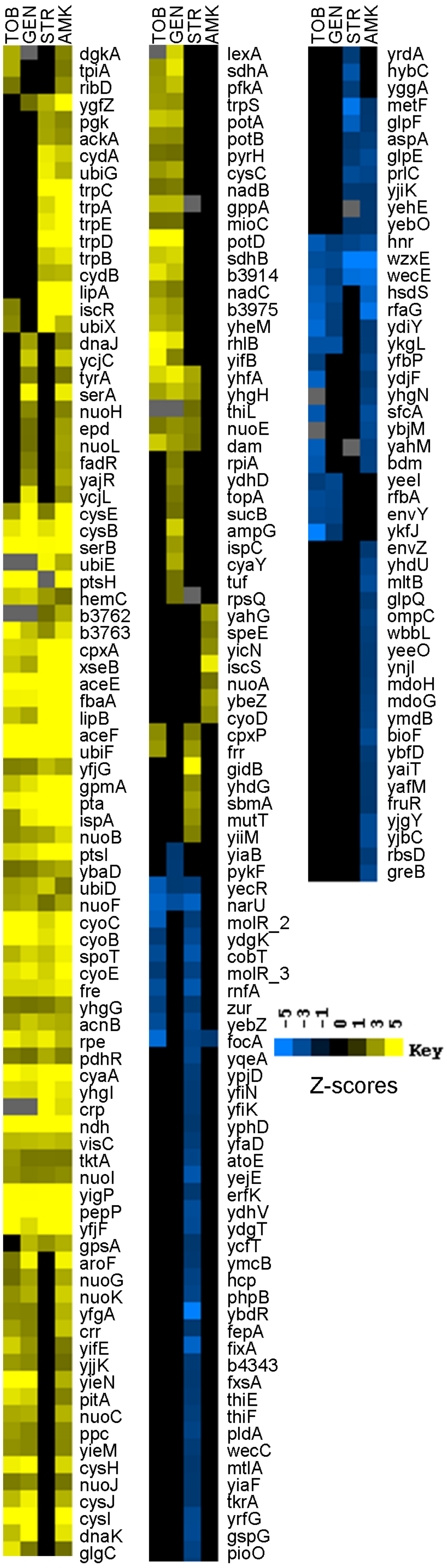

Figure S11. Loci whose disruption was significant in at least one aminoglycoside.
Due to the large size of the set, genes whose disruption was only significant in tobramycin are not shown. Data for tobramycin is available in Dataset S1. Yellow (blue) indicates that transposon insertions in or near a gene were beneficial (deleterious). Black indicates no significant effect; gray indicates missing data.
