# Supplementary material for: Genetic Architecture of Intrinsic Antibiotic Susceptibility
Source: PLoS One. 2009 May 20;4(5):e5629. doi: 10.1371/journal.pone.0005629 (PMC2680486; doi:10.1371/journal.pone.0005629)
Supplement: Table S1 — Loci that changed susceptibility to all aminoglycosides tested. (0.08 MB PDF) [file pone.0005629.s013.pdf]

Table S1. Loci that changed susceptibility to all aminoglycosides tested.

bnum	Effect	Annotation	Comment	References
b0295	down	*ykgL* predicted protein		
b0904	down	*focA* formate FNT transporter		
b1235	down	*rssB* regulator of RpoS		
b1479	down	*maeA* malate dehydrogenase, NAD-requiring		
b1481	down	*bdm* biofilm-dependent modulation protein		
b1626	down	*ydgK* conserved inner membrane protein		
b1627	down	*rsxA* integral membrane protein of SoxR-reducing complex		
b1770	down	*ydjF* predicted DNA-binding transcriptional regulator		
b1825	down	*yebO* predicted protein		
b1840	down	*yebZ* putative resistance protein		
b1976	down	*mtfA* Mlc titration factor		
b2275	down	*yfbP* predicted protein		
b3498	down	*prlC* oligopeptidase A		
b4348	down	*hsdS* specificity determinant for *hsdM* and *hsdR*		
b0014	up	*dnaK* chaperone Hsp70		
b0113	up	*pdhR* transcriptional dual regulator	Signal likely came either from the neighboring gene or from insertions in the gene's pyruvate-binding domain. Deletion of the complete gene should increase NADH levels and increase susceptibility to aminoglycosides.	
b0114	up	*aceE* subunit of E1p component of pyruvate dehydrogenase complex		
b0115	up	*aceF* AceF-lipoate		[1]
b0118	up	*acnB* aconitase B		[2]
b0171	up	*pyrH* uridylate kinase		
b0172	up	*frr* ribosome recycling factor		
b0413	up	*nrdR* DNA-binding transcriptional regulator	Signal likely came from neighboring gene.	
b0414	up	*ribD* fused diaminohydroxyphosphoribosylaminopyrimidine deaminase / 5-amino-6-(5-phosphoribosylamino) uracil reductase		
b0421	up	*ispA* geranyl diphosphate synthase / farnesyl diphosphate synthase		
b0422	up	*xseB* exonuclease VII, small subunit		
b0427	up	*yajR* YajR MFS transporter		
b0428	up	*cyoE* heme O synthase		[2,3]
b0430	up	*cyoC* cytochrome bo terminal oxidase subunit III		
b0431	up	*cyoB* cytochrome bo terminal oxidase subunit I		
b0628	up	*lipA* lipoate synthase monomer		[2]
b0630	up	*lipB* lipoyl-protein ligase		

b0662	up	*ubiF* 2-octaprenyl-3-methyl-6-methoxy-1,4-benzoquinone hydroxylase		[2]
b0724	up	*sdihB* succinate dehydrogenase iron-sulfur protein		[2]
b0755	up	*gpmA* phosphoglyceromutase 1 monomer		
b1109	up	*ndh* NADH dehydrogenase II		
b1187	up	*fadR* FadR transcriptional dual regulator		
b1275	up	*cysB* CysB-O-acetyl-L-serine		
b2232	up	*ubiG* bifunctional 3-demethylubiquinone-8 3-O-methyltransferase and 2-octaprenyl-6-hydroxyphenol methylase		[2]
b2278	up	*nuoL* NADH dehydrogenase I		[2,3]
b2279	up	*nuoK* NADH dehydrogenase I		[2]
b2280	up	*nuoJ* NADH dehydrogenase I		[2,3]
b2281	up	*nuoI* NADH dehydrogenase I		[3]
b2282	up	*nuoH* NADH dehydrogenase I		[2,3]
b2283	up	*nuoG* NADH dehydrogenase I		[2,3]
b2284	up	*nuoF* NADH dehydrogenase I		[2,3]
b2285	up	*nuoE* NADH dehydrogenase I		[2,3]
b2286	up	*nuoC* NADH dehydrogenase I		[2,3]
b2287	up	*nuoB* NADH dehydrogenase I		[3]
b2297	up	*pta* phosphate acetyltransferase		
b2311	up	*ubiX* 3-octaprenyl-4-hydroxybenzoate decarboxylase together with UbiG		[2]
b2416	up	*ptsI* PTS enzyme I		
b2417	up	*crr* N-acetylmuramic acid PTS permease		
b2516	up	*yfgA* putative membrane protein		
b2531	up	*iscR* transcriptional dual regulator		
b2601	up	*aroF* 2-dehydro-3-deoxyphosphoheptonate aldolase		
b2618	up	*yfjF* predicted protein		[2]
b2619	up	*yfjG* toxin of a putative toxin-antitoxin pair		[2]
b2898	up	*ygfZ* folate-binding protein		[2]
b2906	up	*visC* predicted oxidoreductase, FAD/NAD(P)-binding domain	Signal likely came from b2906 (*ubiH*), which was not represented on the array.	
b2908	up	*pepP* proline aminopeptidase P II	Signal likely came from b2906 (*ubiH*), which was not represented on the array.	
b2913	up	*serA* alpha;-ketoglutarate reductase / D-3-phosphoglycerate dehydrogenase		
b2925	up	*fbaA* fructose bisphosphate aldolase monomer		
b2926	up	*pgk* phosphoglycerate kinase		
b2927	up	*epd* erythrose 4-phosphate dehydrogenase		[4]
b2935	up	*tktA* transketolase I		
b3384	up	*trpS* tryptophanyl-tRNA synthetase	Signal likely came from neighboring gene.	
b3386	up	*rpe* ribulose phosphate 3-epimerase		

b3387	up	*dam* DNA adenine methylase	Signal likely came from neighboring gene.	[2]
b3410	up	*feoC* conserved protein		
b3413	up	*gntX* protein involved in utilization of DNA as a carbon source	Signal likely came from neighboring gene.	
b3414	up	*gntY* protein involved in utilization of DNA as a carbon source		
b3607	up	*cysE* serine acetyltransferase monomer		[2,3]
b3608	up	*gpsA* glycerol-3-phosphate-dehydrogenase-[NAD+]		
b3650	up	*spoT* GDP diphosphokinase / guanosine-3',5'-bis(diphosphate) 3'-diphosphatase		
b3745	up	*viaA* stimulator of RavA ATPase activity		
b3746	up	*ravA* regulatory ATPase		
b3763	up	*hdfR* transcriptional repressor		
b3805	up	*hemC* hydroxymethylbilane synthase		
b3834	up	*yigP* conserved protein		
b3843	up	*ubiD* 3-octaprenyl-4-hydroxybenzoate carboxy-lyase monomer		
b3844	up	*fre* FMN reductase		
b3911	up	*cpxA* sensor kinase-phosphotransferase		[5]
b3913	up	*cpxP* regulator of the Cpx response and possible chaperone involved in resistance to extracytoplasmic stress		[2,5]
b3914	up	Obsolete	Signal likely came from neighboring gene.	
b3919	up	*tpiA* triose phosphate isomerase monomer		
b3975	up	no longer thought to be a gene		
b4388	up	*serB* phosphoserine phosphatase		

"Down" indicates that strains with the locus disrupted were depleted during enrichments in aminoglycosides. "Up" indicates that strains with the locus disrupted increased in abundance during the enrichments. See Materials and Methods for details on how the set was identified. In finding references, emphasis was placed on global studies and work in *E. coli*. Expression changes in response to drug addition were not sufficient for inclusion.

References

1. Kohanski MA, Dwyer DJ, Wierzbowski J, Cottarel G, Collins JJ (2008) Mistranslation of membrane proteins and two-component system activation trigger antibiotic-mediated cell death. Cell 135: 679-690.
2. Kohanski MA, Dwyer DJ, Hayete B, Lawrence CA, Collins JJ (2007) A common mechanism of cellular death induced by bactericidal antibiotics. Cell 130: 797-810.
3. Schurek KN, Marr AK, Taylor PK, Wiegand I, Semenec L, et al. (2008) Novel genetic determinants of low-level aminoglycoside resistance in *Pseudomonas aeruginosa*. Antimicrob Agents Chemother 52: 4213-4219.
4. Fajardo A, Martinez-Martin N, Mercadillo M, Galan JC, Ghysels B, et al. (2008) The neglected intrinsic resistome of bacterial pathogens. PLoS ONE 3: e1619.
5. Rainwater S, Silverman PM (1990) The Cpx proteins of *Escherichia coli* K-12: evidence that *cpxA*, *ecfB*, *ssd*, and *eup* mutations all identify the same gene. J Bacteriol 172: 2456-2461.
