# Supplementary material for: Genetic Architecture of Intrinsic Antibiotic Susceptibility
Source: PLoS One. 2009 May 20;4(5):e5629. doi: 10.1371/journal.pone.0005629 (PMC2680486; doi:10.1371/journal.pone.0005629)
Supplement: Table S2 — Loci that changed susceptibility to all beta-lactams tested. (0.07 MB PDF) [file pone.0005629.s014.pdf]

Table S2. Loci that changed susceptibility to all β-lactams tested.

bnum	Effect	Annotation	References
b0196	down	*rcsF* phosphorelay glucose and zinc sensor	[1]
b0433	down	*ampG* muropeptide MFS transporter	[2]
b2317	down	*dedA* conserved inner membrane protein	
b2701	down	*mltB* membrane-bound lytic murein transglycosylase B	
b2786	down	*barA*	
b3013	down	*yqhG* conserved protein	
b3234	down	*degQ* serine endoprotease, periplasmic	
b3388	down	*damX* predicted membrane-anchored protein, interferes with cell division	
b3753	down	*rbsR* transcriptional repressor	
b3793	down	*wzyE* predicted Wzy protein involved in ECA polysaccharide chain elongation	
b4150	down	*ampC* beta;-lactamase; penicillin resistance	[3]
b4392	down	*slt* soluble lytic murein transglycosylase	
b0439	up	*lon* DNA-binding, ATP-dependent protease	
b0888	up	*trxB* thioredoxin reductase monomer	
b0928	up	*aspC* aspartate aminotransferase, PLP-dependent	
b1071	up	*flgM* anti-sigma factor for FliA (sigma 28)	
b1074	up	*flgC* flagellar basal-body rod protein	
b1075	up	*flgD* flagellar biosynthesis, initiation of hook assembly	
b1076	up	*flgE* flagellar hook protein	
b1077	up	*flgF* flagellar basal-body rod protein	
b1078	up	*flgG* flagellar basal-body rod protein	
b1292	up	*sapC* peptide uptake ABC transporter	
b1677	up	*lpp* murein lipoprotein	
b1830	up	*prc* tail-specific protease	
b1879	up	*flhA* flagellar biosynthesis protein	
b1880	up	*flhB* flagellar biosynthesis protein	
b1891	up	*flhC* FlhDC transcriptional dual regulator	
b1892	up	*flhD* FlhDC transcriptional dual regulator	
b1921	up	*fliZ* predicted regulator of FliA activity	
b1922	up	*fliA* RNA polymerase, sigma 28 (sigma F) factor	
b1939	up	*fliG* flagellar motor switch protein	
b1940	up	*fliH* flagellar biosynthesis protein	
b1941	up	*fliI* flagellum-specific ATP synthase	
b1946	up	*fliN* flagellar motor switch protein	
b1950	up	*fliR* flagellar biosynthesis protein	
b2029	up	*gnd* 6-phosphogluconate dehydrogenase (decarboxylating)	
b2066	up	*udk* uridine kinase / cytidine kinase	
b2143	up	*cdd* cytidine deaminase	
b2216	up	*rcsD* putative 2-component sensor protein	[1]
b2684	up	*mprA* transcriptional repressor	[4]
b2903	up	*gcvP* glycine decarboxylase	
b2905	up	*gcvT* aminomethyltransferase	
b3210	up	*arcB* aerobic respiration control sensor protein	[5]
b3408	up	*feoA* ferrous iron transport protein A	
b3843	up	*ubiD* 3-octaprenyl-4-hydroxybenzoate carboxy-lyase monomer	

"Down" indicates that strains with the locus disrupted were depleted during enrichments in β-lactams. "Up" indicates that strains with the locus disrupted increased in abundance during the enrichments. See Materials and Methods for details on how the set was identified. In finding references, emphasis was placed on global studies and work in *E. coli*. Expression changes in response to drug addition were not sufficient for inclusion.

References

1. Laubacher ME, Ades SE (2008) The Rcs phosphorelay is a cell envelope stress response activated by peptidoglycan stress and contributes to intrinsic antibiotic resistance. J Bacteriol 190: 2065-2074.
2. Jacobs C, Huang LJ, Bartowsky E, Normark S, Park JT (1994) Bacterial cell wall recycling provides cytosolic muropeptides as effectors for beta-lactamase induction. Embo J 13: 4684-4694.
3. Mazzariol A, Cornaglia G, Nikaido H (2000) Contributions of the AmpC beta-lactamase and the AcrAB multidrug efflux system in intrinsic resistance of *Escherichia coli* K-12 to beta-lactams. Antimicrob Agents Chemother 44: 1387-1390.
4. Lomovskaya O, Lewis K, Matin A (1995) EmrR is a negative regulator of the *Escherichia coli* multidrug resistance pump EmrAB. J Bacteriol 177: 2328-2334.
5. Okusu H, Ma D, Nikaido H (1996) AcrAB efflux pump plays a major role in the antibiotic resistance phenotype of *Escherichia coli* multiple-antibiotic-resistance (Mar) mutants. J Bacteriol 178: 306-308.
