# Supplementary material for: Genetic Architecture of Intrinsic Antibiotic Susceptibility
Source: PLoS One. 2009 May 20;4(5):e5629. doi: 10.1371/journal.pone.0005629 (PMC2680486; doi:10.1371/journal.pone.0005629)
Supplement: Table S3 — Genes identified in this work as having a general role in antibiotic susceptibility. (0.07 MB PDF) [file pone.0005629.s015.pdf]

Table S3. Genes identified in this work as having a general role in antibiotic susceptibility.

bnum	Annotation	References
b3791	*rffA* dTDP-4-oxo-6-deoxy-D-glucose transaminase	[1]
b3792	*wzxE* lipid III flippase	
b1904	*yecR* predicted protein	
b2616	*recN* protein used in recombination and DNA repair	[1,2,3,4]
b2494	*yfgC* predicted peptidase	[1,5]
b0462	*acrB* RND-type permease	[1,6,7]
b1130	*phoP* PhoP-Phosphorylated transcriptional dual regulator	[1]
b4396	*rob* Rob transcriptional activator	[8]
b3194	*yrbE* YrbF/YrbE ABC transporter	
b3191	*yrbB* predicted NTP-binding protein	
b3632	*rfaQ* lipopolysaccharide core biosynthesis protein; heptosyl transferase III	[9]
b3630	*rfaP* lipopolysaccharide core biosynthesis; phosphorylation of core heptose	[9]
b3628	*rfaB* UDP-D-galactose:(glucosyl)lipopolysaccharide-1,6-D-galactosyltransferase	[9]
b3631	*rfaG* lipopolysaccharide glucosyltransferase I	[9]
b2215	*ompC* outer membrane porin C	
b3780	*rhlB* degradosome	
b3956	*ppc* phosphoenolpyruvate carboxylase	
b3493	*pitA* PitA	
b2415	*ptsH* HPr	
b3806	*cyaA* adenylate cyclase	[10]
b0015	*dnaJ* chaperone with DnaK; heat shock protein	[11]
b1048	*mdoG* periplasmic glucan (MDO) biosynthesis protein	
b1049	*mdoH* membrane glycosyltransferase; synthesis of membrane-derived oligosaccharide (MDO)	
b2218	*rcsC* RcsC-P his	[12]
b1291	*sapD* peptide uptake ABC transporter	
b3290	*trkA* NAD-binding component of TrK potassium transporter	
b1290	*sapF* peptide uptake ABC transporter	
b3849	*trkH* TrkH potassium ion Trk Transporter	
b0464	*acrR* AcrR transcriptional repressor	[1,13]
b1676	*pykF* pyruvate kinase I monomer	

In finding references, emphasis was placed on global studies and work in *E. coli*. Expression changes in response to drug addition were not sufficient for inclusion. Results and Discussion describes how the class of genes was determined.

References

1. Tamae C, Liu A, Kim K, Sitz D, Hong J, et al. (2008) Determination of antibiotic hypersensitivity among 4,000 single-gene-knockout mutants of *Escherichia coli*. J Bacteriol 190: 5981-5988.
2. Walters RN, Piddock LJ, Wise R (1989) The effect of mutations in the SOS response on the kinetics of quinolone killing. J Antimicrob Chemother 24: 863-873.
3. Sengupta S, Rahman MS, Mukherjee U, Basak J, Pal AK, et al. (1990) DNA damage and prophage induction and toxicity of nitrofurantoin in *Escherichia coli* and *Vibrio cholerae* cells. Mutat Res 244: 55-60.

4. Kosa JL, Zdraveski ZZ, Currier S, Marinus MG, Essigmann JM (2004) RecN and RecG are required for *Escherichia coli* survival of Bleomycin-induced damage. Mutat Res 554: 149-157.
5. Fajardo A, Martinez-Martin N, Mercadillo M, Galan JC, Ghysels B, et al. (2008) The neglected intrinsic resistome of bacterial pathogens. PLoS ONE 3: e1619.
6. Gomez MJ, Neyfakh AA (2006) Genes involved in intrinsic antibiotic resistance of *Acinetobacter baylyi*. Antimicrob Agents Chemother 50: 3562-3567.
7. Okusu H, Ma D, Nikaido H (1996) AcrAB efflux pump plays a major role in the antibiotic resistance phenotype of *Escherichia coli* multiple-antibiotic-resistance (Mar) mutants. J Bacteriol 178: 306-308.
8. Rosenberg EY, Bertenthal D, Nilles ML, Bertrand KP, Nikaido H (2003) Bile salts and fatty acids induce the expression of *Escherichia coli* AcrAB multidrug efflux pump through their interaction with Rob regulatory protein. Mol Microbiol 48: 1609-1619.
9. Hancock RE (1984) Alterations in outer membrane permeability. Annu Rev Microbiol 38: 237-264.
10. Alper MD, Ames BN (1978) Transport of antibiotics and metabolite analogs by systems under cyclic AMP control: positive selection of *Salmonella typhimurium cya* and *crp* mutants. J Bacteriol 133: 149-157.
11. Wolska KI, Bugajska E, Jurkiewicz D, Kuc M, Jozwik A (2000) Antibiotic susceptibility of *Escherichia coli dnaK* and *dnaJ* mutants. Microb Drug Resist 6: 119-126.
12. Laubacher ME, Ades SE (2008) The Rcs phosphorelay is a cell envelope stress response activated by peptidoglycan stress and contributes to intrinsic antibiotic resistance. J Bacteriol 190: 2065-2074.
13. Ma D, Alberti M, Lynch C, Nikaido H, Hearst JE (1996) The local repressor AcrR plays a modulating role in the regulation of *acrAB* genes of *Escherichia coli* by global stress signals. Mol Microbiol 19: 101-112.
