# Supplementary material for: Genetic Architecture of Intrinsic Antibiotic Susceptibility
Source: PLoS One. 2009 May 20;4(5):e5629. doi: 10.1371/journal.pone.0005629 (PMC2680486; doi:10.1371/journal.pone.0005629)
Supplement: Table S4 — Additional genes identified in both this study and previous work. (0.07 MB PDF) [file pone.0005629.s016.pdf]

Table S4. Additional genes identified in both this study and previous work.

bnum	Annotation	Effect	Notes	Drug	Reference
b4058	*uvrA* excision nuclease subunit A	down		NIT	[1]
b3822	*recQ* ATP-dependent DNA helicase	down		NIT	[1]
b0851	*nfsA* NADPH nitroreductase	up		NIT	[2]
b0850	*ybjC* predicted inner membrane protein	up	Effect is likely polar on b0851.	NIT	[2]
b1861	*ruvA* branch migration of Holliday structures; repair	down		NAL, LOM	[3]
b0929	*ompF* The Colicin A Import System	up		TET	[4]
b0888	*trxB* thioredoxin reductase monomer	up		DOX, TET	[5]

"Down" indicates that strains with the locus disrupted were depleted during enrichments in the indicated drug(s). "Up" indicates that strains with the locus disrupted increased in abundance during the enrichments. In finding references, emphasis was placed on global studies and work in *E. coli*. Expression changes in response to drug addition were not sufficient for inclusion.

References

1. Sengupta S, Rahman MS, Mukherjee U, Basak J, Pal AK, et al. (1990) DNA damage and prophage induction and toxicity of nitrofurantoin in *Escherichia coli* and *Vibrio cholerae* cells. Mutat Res 244: 55-60.
2. Whiteway J, Koziarz P, Veall J, Sandhu N, Kumar P, et al. (1998) Oxygen-insensitive nitroreductases: analysis of the roles of *nfsA* and *nfsB* in development of resistance to 5-nitrofuran derivatives in *Escherichia coli*. J Bacteriol 180: 5529-5539.
3. Walters RN, Piddock LJ, Wise R (1989) The effect of mutations in the SOS response on the kinetics of quinolone killing. J Antimicrob Chemother 24: 863-873.
4. Thanassi DG, Suh GS, Nikaido H (1995) Role of outer membrane barrier in efflux-mediated tetracycline resistance of *Escherichia coli*. J Bacteriol 177: 998-1007.
5. Fajardo A, Martinez-Martin N, Mercadillo M, Galan JC, Ghysels B, et al. (2008) The neglected intrinsic resistome of bacterial pathogens. PLoS ONE 3: e1619.
