# Supplementary material for: Genetic Architecture of Intrinsic Antibiotic Susceptibility
Source: PLoS One. 2009 May 20;4(5):e5629. doi: 10.1371/journal.pone.0005629 (PMC2680486; doi:10.1371/journal.pone.0005629)
Supplement: Table S5 — MIC changes in aminoglycosides. (0.06 MB PDF) [file pone.0005629.s017.pdf]

**Table S5. MIC changes in aminoglycosides.**

| | Relative Susceptibility[a] / Change in Enrichment[b] | | | |
|---|---|---|---|---|
| NAME | AMK | GEN | STR | TOB |
| *acnB::kan* | 1 / ↑ | -1 / ↑ | 0 / ↑ | 0 / ↑ |
| *sbmA::kan* | 1 / ↔ | 0 / ↔ | 1 / ↑ | -1 / ↔ |
| *cyoA::kan* | 2 / ND | 1 / ND | 2 / ND | 1 / ↑ |
| *ubiF::kan* | 3 / ↑ | 1 / ↑ | 2 / ↑ | 2 / ↑ |
| *puuR::kan* | 1 / ↑ | 0 / ↑ | 1 / ↔ | 0 / ↔ |
| *nuoG::kan* | 0 / ↑ | 0 / ↑ | 1 / ↑ | 0 / ↑ |
| *gpp::kan*[c] | 2 / ↔ | 2 / ↑ | 1 / ↔ | 1 / ↑ |
| *cyaA::kan* | 3 / ↑ | 1 / ↑ | 2 / ↑ | 0 / ↑ |
| *cpxA::kan* | 2 / ↑ | 2 / ↑ | 0 / ↑ | 1 / ↑ |

[a] Relative susceptibility is expressed as $\log_{1.5}$(MIC mutant)/(MIC wild-type), where MIC is the minimal inhibitory concentration for the given antibiotic.

[b] A "↑" ("↓") indicates that mutants with transposons in or near the locus were significantly enriched (depleted) during growth in the indicated antibiotic; "↔" indicates that no significant change occurred.

[c] MIC was determined after 48 hours. All other measurements were after 24 hours.

ND: no data
