# Supplementary material for: Genetic Architecture of Intrinsic Antibiotic Susceptibility
Source: PLoS One. 2009 May 20;4(5):e5629. doi: 10.1371/journal.pone.0005629 (PMC2680486; doi:10.1371/journal.pone.0005629)
Supplement: Table S6 — Additional class-specific MIC changes (non-aminoglycosides). (0.07 MB PDF) [file pone.0005629.s018.pdf]

Table S6. Additional class-specific MIC changes (non-aminoglycosides).

	Relative Susceptibility[a] / Change in Enrichment[b]						
Genotype	DOX	TET	LOM	NAL	BLM	SLF	TRM
lon::kan	1 / ↑	1 / ↑	ND / ↔	ND / ↔	ND / ↔	ND / ↔	ND / ↔
ppk::kan	1 / ↑	1 / ↑	-1 / ↔	1 / ↑	ND / ↔	ND / ↔	ND / ↔
rseB::kan	-1 / ↓	0 / ↔	ND / ↔	ND / ↔	ND / ↔	ND / ↔	ND / ↔
qseB::kan	<-2 / ↓	0 / ↔	-1 / ↔	-2 / ↓	ND / ↔	ND / ↔	ND / ↔
ompR::kan	0 / ↔	1 / ↑	ND / ↔	ND / ↔	ND / ↔	ND / ↔	ND / ↔
sbmA::kan	ND / ↔	ND / ↔	ND / ↔	ND / ↔	2 / ↑	ND / ↔	ND / ↔
prlC::kan	ND / ↔	ND / ↔	ND / ↔	ND / ↔	-1 / ND	ND / ↔	ND / ↔
folM::kan	ND / ↔	ND / ↔	ND / ↔	ND / ↔	ND / ↔	4 / ↑	0 / ↑
folX::kan	ND / ↔	ND / ↔	ND / ↔	ND / ↔	ND / ↔	2 / ↔	0 / ↑
yafD::kan	ND / ↔	ND / ↔	ND / ↔	ND / ↔	ND / ↔	ND / ↔	1 / ↑

[a] Relative susceptibility is expressed as $\log_{1.5}$(MIC mutant)/(MIC wild-type), where MIC is the minimal inhibitory concentration for the given antibiotic.
[b] A "↑" ("↓") indicates that mutants with transposons in or near the locus were significantly enriched (depleted) during growth in the indicated antibiotic; "↔" indicates that no significant change occurred. ND: no data
