# Supplementary material for: Genetic Architecture of Intrinsic Antibiotic Susceptibility
Source: PLoS One. 2009 May 20;4(5):e5629. doi: 10.1371/journal.pone.0005629 (PMC2680486; doi:10.1371/journal.pone.0005629)
Supplement: Table S7 — MIC changes for mutants with altered susceptibility to multiple drug classes. (0.07 MB PDF) [file pone.0005629.s019.pdf]

Table S7. MIC changes for mutants with altered susceptibility to multiple drug classes.

	Relative Susceptibility[a] / Change in Enrichment[b]								
Genotype	AMK	AMP	BLM	DOX	FUS	LOM	NAL	TET	TRM
phoP::kan	ND	-1 / ↓	ND / ↔	-5 / ↓	ND / ↔	-1 / ↓	-1 / ↓	-1 / ↓	-1 / ↓
sapD::kan	ND	ND / ↔	ND / ↔	1 / ↑	ND / ↔	-1 / ↔	1 / ↑	1 / ↑	ND / ↔
yrbE::kan	ND	ND / ↔	1 / ↑	>-5 / ↓	ND / ↔	-1 / ↓	-2 / ↓	0 / ↔	ND / ↔
rfaG::kan	0 / ↓	1 / ↔	ND / ↔	-4 / ↓	ND / ↔	-1 / ↓	-4 / ↓	0 / ↔	-3 / ↓
yfgC::kan	1 / ↔	-1 / ↓	ND / ↓	-1 / ↓	-4 / ↓	0 / ↔	-1 / ↓	0 / ↔	-1 / ↔
wzxE::kan	-2 / ↓	0 / ↔	ND / ↓	0 / ↑	ND / ↔	0 / ↔	-1 / ↓	0 / ↔	0 / ↔
ompC::kan	0 / ↓	0 / ↓	ND / ↔	0 / ↓	ND / ↔	0 / ↓	-1 / ↓	0 / ↔	0 / ↔

[a] Relative susceptibility is expressed as $\log_{1.5}$(MIC mutant)/(MIC wild-type), where MIC is the minimal inhibitory concentration for the given antibiotic.
[b] A "↑" ("↓") indicates that mutants with transposons in or near the locus were significantly enriched (depleted) during growth in the indicated antibiotic; "↔" indicates that no significant change occurred.
ND: no data
